# Multi‐omics analysis reveals the association between specific solute carrier proteins gene expression patterns and the immune suppressive microenvironment in glioma

**DOI:** 10.1111/jcmm.18339

**Published:** 2024-04-30

**Authors:** Wenjie Wu, Cheng Jiang, Wende Zhu, Xiaobing Jiang

**Affiliations:** ^1^ Department of Neurosurgery, Union Hospital, Tongji Medical College Huazhong University of Science and Technology Wuhan China

**Keywords:** bulk RNA‐seq, glioma, single‐cell RNA‐seq, SLC43A3, solute carrier proteins, tumour microenvironment, tumour‐associated macrophages

## Abstract

Glioma is the most prevalent malignant brain tumour. Currently, reshaping its tumour microenvironment has emerged as an appealing strategy to enhance therapeutic efficacy. As the largest group of transmembrane transport proteins, solute carrier proteins (SLCs) are responsible for the transmembrane transport of various metabolites and ions. They play a crucial role in regulating the metabolism and functions of malignant cells and immune cells within the tumour microenvironment, making them a promising target in cancer therapy. Through multidimensional data analysis and experimental validation, we investigated the genetic landscape of SLCs in glioma. We established a classification system comprising 7‐SLCs to predict the prognosis of glioma patients and their potential responses to immunotherapy and chemotherapy. Our findings unveiled specific SLC expression patterns and their correlation with the immune‐suppressive microenvironment and metabolic status. The 7‐SLC classification system was validated in distinguishing subgroups within the microenvironment, specifically identifying subsets involving malignant cells and tumour‐associated macrophages. Furthermore, the orphan protein SLC43A3, a core member of the 7‐SLC classification system, was identified as a key facilitator of tumour cell proliferation and migration, suggesting its potential as a novel target for cancer therapy.

## 1 INTRODUCTION

Glioma is the most prevalent malignant primary brain tumour, accounting for approximately 81% of all malignant brain tumours, with a 5‐year overall survival rate of approximately 35%.^1^, ^2^, ^3^ Based on the pathological histology, gliomas are categorized into WHO grades I‐IV, where glioblastoma (GBM, WHO IV) represents the most common histological subtype, constituting about 45% of all gliomas, and exhibiting a 5‐year survival rate of only about 5%.^1^, ^2^, ^3^, ^4^, ^5^ Despite significant advances in surgical, radiotherapeutic, chemotherapeutic and other novel therapeutic modalities in recent years, there has been limited improvement in the prognosis of glioblastoma patients.^6^, ^7^, ^8^ Recently, with the rapid development of single‐cell and spatial transcriptomics, substantial progress has been made in understanding the complex tumour microenvironment of glioblastoma. Current research has revealed intricate interactions and heterogeneity among cellular components, including tumour cells, tumour‐associated macrophages (TAMs) and T cells, leading to the proposal of novel therapeutic approaches for reshaping the glioblastoma tumour microenvironment, which include reprogramming TAMs to counteract immune suppression, preventing T‐cell exhaustion and targeting tumour metabolism.^9^, ^10^, ^11^ These novel therapeutic concepts have demonstrated promising outcomes in preclinical trials.^9^, ^10^, ^11^, ^12^ Therefore, identifying new molecular targets capable of reshaping the immune‐suppressive microenvironment in glioblastoma holds the potential to bring new hope to tumour treatment.

Solute carrier proteins (SLCs) constitute the largest group of transmembrane transporters, facilitating the transmembrane transport of nutrients, metal ions, metabolic byproducts, drugs and various other substances.^13^, ^14^ During the process of tumour progression, tumour cells undergo alterations in their metabolism, leading to an increased demand for energy and essential nutrients, including carbohydrates, proteins, lipids and nucleic acids. The expression of SLCs, which serve as the primary transporters for these molecules, often undergoes changes.^15^ Hence, targeting these SLCs has long been considered a promising therapeutic approach for cancer.^15^, ^16^ In recent years, SLCs have also been discovered to play a crucial role in regulating the metabolic phenotypes of immune cells.^17^, ^18^ Immune cells can induce rapid and robust metabolic reprogramming by modifying specific SLC expression patterns, thereby regulating different immune responses, making SLCs pivotal in the context of cancer immunotherapy.^18^ However, the role of SLCs in gliomas regarding tumour progression, immune regulation and microenvironment shaping has not been thoroughly investigated, thus highlighting an urgent need for research into the potential roles of SLCs in gliomas.

**FIGURE 1:**
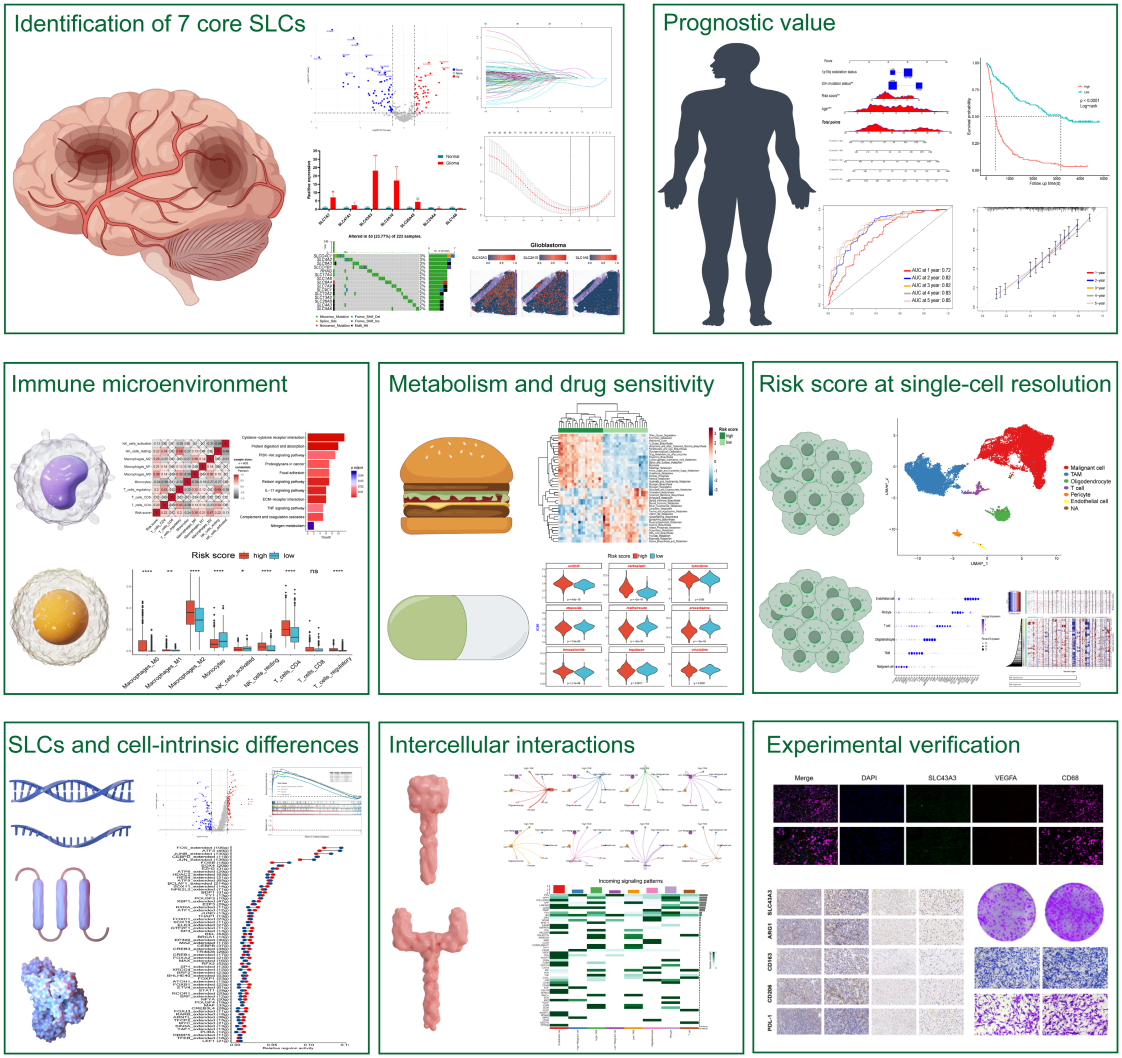
Graphical abstract of this study.

In this study, comprehensive bioinformatics analysis of the SLC gene family was conducted based on multi‐omics and multidimensional data, with the aim of uncovering SLC molecules within gliomas that hold potential for prognosis and therapeutic significance. We explored the genetic landscape of SLCs within gliomas and established a model based on the expression of seven core SLCs that can predict patient prognosis and their potential response to immunotherapy or chemotherapy. Furthermore, we conducted a detailed single‐cell resolution analysis of the distinct expression patterns of seven key SLCs, exploring their connections with cellular metabolism, transcriptional regulation and intercellular interactions. Finally, we substantiated the crucial role of the model's core member, SLC43A3, in promoting the malignant phenotypes of glioblastoma cells, including proliferation and migration through molecular biology experiments. Additionally, we provided evidence for the correlation between the expression patterns of specific SLCs and the immunosuppressive microenvironment in glioma. It is our aspiration that our research on SLCs in glioma can offer fresh insights into reshaping the tumour microenvironment and assist in the development of novel targeted therapeutic approaches. The graphical abstract of this study is shown in Figure 1.

## 2 MATERIALS AND METHODS

### 2.1 Data collection

The copy number variation (CNV), single nucleotide variant, gene methylation, transcriptomic and corresponding clinical information for the TCGA pan‐glioma cohort were obtained from The Cancer Genome Atlas (TCGA, https://portal.gdc.cancer.gov/). The transcriptomic data and corresponding clinical information for the external validation cohorts, CGGA693 and CGGA325, were downloaded from the CGGA database (http://www.cgga.org.cn/). The gene expression microarray data (GSE50161), single‐cell sequencing data for seven glioblastoma samples (GSE135045) and spatial transcriptomic data (GSE194329) were obtained from the GEO database (https://www.ncbi.nlm.nih.gov/geo/). The detailed information on the 368 SLCs included in the study was obtained from previous research literature,^14^ as outlined in Table S1. Samples lacking the corresponding clinical information were excluded from our study. Ultimately, our study included 152 GBM samples and 451 LGG samples from the TCGA pan‐glioma cohort, 137 GBM samples and 176 LGG samples from the CGGA325 cohort, and 237 GBM samples and 420 LGG samples from the CGGA693 cohort.

### 2.2 Establishment and validation of a risk scoring system based on seven SLC genes

To identify differentially expressed SLC genes in glioblastoma, the R package Limma^19^ was employed to conduct differential expression analysis on the GSE50161 dataset, which includes gene expression data from 34 glioblastoma samples and 13 normal brain tissue samples. Adjusted *p*‐values below 0.05 and logFC greater than 2 or less than 0.5 were used as criteria to confirm differentially expressed SLC genes. In the TCGA pan‐glioma cohort, prognosis‐related SLC genes were initially selected through univariate Cox analysis (*p* < 0.05). Subsequently, utilizing the R package glmnet,^20^ differentially expressed SLC genes with prognostic value were further filtered using the LASSO‐COX algorithm, resulting in the identification of seven core SLC genes: SLC43A3, SLC2A10, SLC25A43, SLC7A7, SLC47A1, SLC1A6 and SLC24A4. The final risk score for each patient is determined using the following formula: $\text{Risk score}=\sum_{n=1}^{7}\left(\exp_{n}*{\text{coef}}_{n}\right)$, where exp is the expression of SLCs and coef is the regression coefficient.

According to the aforementioned formula, the risk scores of patients from three independent datasets, TCGA, CGGA325 and CGGA693, were calculated. Based on the median risk score, each dataset's patients were categorized into high‐risk and low‐risk groups. Subsequently, the R packages survival and survminer were employed to conduct Kaplan–Meier survival analysis, assessing differences in survival rates between the high‐risk and low‐risk groups. Additionally, the R package timeROC was utilized to perform time‐dependent ROC curve analysis to determine the accuracy and specificity of risk score predictions for prognosis.

### 2.3 Construction and validation of a nomogram in glioma

In conjunction with other prognostic clinical and pathological features, such as age, IDH mutation status and 1p19q co‐deletion status, the R package regplot was utilized to construct a nomogram for predicting the prognosis of glioma patients. Furthermore, to facilitate the use of the nomogram, a user‐friendly dynamic nomogram was constructed using the shinyapps platform (https://www.shinyapps.io/). The dynamic nomogram can be accessed via the following link: (https://net‐visualization.shinyapps.io/Nomogram/). Time‐dependent receiver operating characteristic curves (Time‐dependent ROC) and calibration curves were employed to assess the performance of the nomogram on both internal and external datasets. Decision curve analysis (DCA) curves were used to evaluate the clinical utility and net benefit of the nomogram.

### 2.4 Analysis of risk‐associated differential genes and functional inference

Based on the median risk score, the TCGA pan‐glioma, CGGA693 and CGGA325 cohorts were stratified into high‐risk and low‐risk groups. The R package DESeq2^21^ was utilized to analyse differential gene expression between these two groups. Subsequently, we conducted enrichment analysis on the overlapping upregulated or downregulated genes in the three cohorts using the R package clusterProfiler,^22^ focusing on three databases: Gene Ontology (GO), Kyoto Encyclopedia of Genes and Genomes (KEGG) and Reactome.

### 2.5 Analysis of tumour immune infiltration and prediction of immunotherapy outcomes

The abundance of immune cell infiltration in tumour samples was analysed using three distinct methods, CIBERSORT,^23^ quanTIseq^24^ and ssGSEA,^25^ through the R package IOBR.^26^ T cells marked with CD8 or CD4 are integrated as CD8+ T cells and CD4+ T cells. The potential response of patients in the high‐ and low‐risk groups to immunotherapy was predicted using the TIDE^27^ algorithm. Three publicly available real‐world immunotherapy datasets, LGG_E‐MTAB‐6270, NSCLC_GSE126044 and RCC_GSE67501, were downloaded from TIGER (http://tiger.canceromics.org/). Risk scores for each patient were calculated based on the previously established formula. ROC curve analysis was used to evaluate the discriminative performance of the risk scores.

### 2.6 Inference of metabolic pathway activity and analysis of potential drug sensitivity

The activity of relevant metabolic pathways in the high‐ and low‐risk groups was calculated using the R package IOBR.^26^ The metabolic pathways were sourced from KEGG, and the computation method employed was ssGSEA. Gene expression and drug sensitivity data for human tumour cell lines were obtained from Genomics of Drug Sensitivity in Cancer and Cancer Therapeutics Response Portal and used as the training dataset. To avoid introducing additional errors, both the training set and our target expression matrix data formats adopted log2‐transformed FPKM values. The R package oncoPredict was utilized to calculate IC50 values for chemotherapy drugs in both high‐ and low‐risk group patients.^28^

### 2.7 Processing workflow for single‐cell and spatial transcriptomes

We processed single‐cell sequencing data (GSE135045) and spatial transcriptome data (GSE194329) using the R package Seurat.^29^ Single‐cell sequencing data underwent preprocessing following previously established methods to filter out low‐quality cells.^30^ The LogNormalize method was used to normalize single‐cell expression data, followed by the selection of 2000 highly variable genes for downstream analysis. The first 25 PCA dimensions corrected by the harmony algorithm were used for Uniform Manifold Approximation and Projection dimensionality reduction. Batch effects between different samples were mitigated using the Harmony algorithm.^31^ Various cell subpopulations were annotated based on their corresponding cell markers, and CNVs in tumour cells were predicted using the R package inferCNV.^32^ The distinct transcriptional features associated with different tumour cell states were based on prior research.^33^ The addModuleScore algorithm was used to calculate scores for four cellular states in malignant cells. The first 25 dimensions corrected by harmony were used for cell clustering, with a resolution selection of 0.05. Cells with scores in the top 25% for a specific cell state were annotated as belonging to that particular cell state. Subsequently, cells with scores in the lower 75% range for all cell states were classified as mixed‐cell states. Employing the formula referenced in the preceding discussion, we quantified the risk scores associated with malignant cells and TAMs. Given the observation that not all solute carrier protein expression value was identified within each cell, a substantial number of cells were assigned a risk score of zero. To enhance the precision of our evaluation, cells exhibiting a risk score exceeding zero were classified as harbouring a high‐risk phenotype. Spatial transcriptome data were normalized using the SCTransform algorithm. We applied the standard processing workflow to three GBM tissue sections and one adjacent non‐cancerous tissue section.

### 2.8 Pseudo‐temporal analysis, cellular communication analysis, transcriptional regulatory network construction and inference of cellular metabolic activity

We employed the R package Monocle3^34^ for pseudo‐temporal analysis to determine the differentiation trajectory of tumour cell subpopulations. The transcriptional regulatory networks of high‐ and low‐risk group cells were constructed utilizing the R package SCENIC.^35^ Subsequently, we calculated the average transcription factor activity for each group. Furthermore, we assessed the metabolic pathway activity of high‐ and low‐risk group cells using the R package scMetabolism,^36^ with metabolic pathway information sourced from the KEGG database and the algorithm we utilized was VISION. The communication networks between different cell populations were constructed using the R package CellChat.^37^ All ligand–receptor pairs in CellChatDB.human were used for analysis. We placed particular emphasis on examining the differences in cellular communication patterns between the high‐ and low‐risk score groups, specifically focusing on malignant cells and TAMs.

### 2.9 Expression level detection

For quantitative real‐time polymerase chain reaction (qPCR), total RNA extraction was performed using the RNAiso Plus (Takara, 9109), according to the manufacturer's instructions. The concentration and purity of the extracted RNA were assessed using a spectrophotometer. Subsequently, cDNA synthesis was carried out using the RT Supermix Reagent Kit (Vazyme, R323). qPCR was conducted using SYBR qPCR Master Mix (Vazyme, Q311‐02/03). Correlation analysis utilized ΔΔCT values of the target gene normalized to the Glyceraldehyde‐3‐phosphate dehydrogenase (GAPDH). For immunohistochemistry, the tissue specimens were initially fixed using a 10% formalin solution and subsequently embedded in paraffin before undergoing sectioning. Deparaffinization and hydration of the sections were then carried out, followed by antigen retrieval in citrate buffer with a pH of 6. To inhibit endogenous peroxidase activity, the sections were treated with methanol containing 3% hydrogen peroxide. To minimize nonspecific staining, tissue sections were incubated with 3% bovine serum albumin. Subsequently, the sections underwent an overnight incubation at 4°C with the primary antibodies. After washing with PBS, secondary antibodies (HRP polymer) were applied, and the sections were incubated at 37°C for 50 min. Diaminobenzidine was then used for visualization, followed by haematoxylin counterstaining and mounting with a cover glass.

### 2.10 Cell functional assay

Cells were cultured in Dulbecco's modified Eagle medium (DMEM) supplemented with 10% foetal bovine serum and 1% penicillin/streptomycin. Gene knockdown and overexpression experiments were carried out via lentiviral transfection of the target cells. Lentiviral vectors, incorporating constructs for gene knockdown (short hairpin RNA) or overexpression, were designed and transfected into the target cells. Puromycin was used to select for stable cell lines. Quantitative polymerase chain reaction (qPCR) was utilized to validate the expression levels of the target gene. Cell viability was assessed using the CCK8 assay. Cells were seeded in a 96‐well plate at a consistent density per well. CCK8 solution was added at specified time points, and absorbance was measured at 450 nm using a microplate reader. For the colony formation assay, cells were plated at a density of 1000 cells per well in DMEM and incubated for 14 days. Colonies were fixed with paraformaldehyde and stained with 0.01% crystal violet. For 5‐ethynyl‐2′‐deoxyuridine (EdU) incorporation assay, cells were labelled with EdU (10 μM) for 2–3 h, fixed with paraformaldehyde and stained using the EdU Kit (Beyotime, C0078S) according to the manufacturer's instructions. Nuclei were stained with Hoechst 33342, and the percentage of EdU‐positive cells was determined. For the transwell assay, cell migration was assessed using transwell chambers with membranes of 8 μm pore size. Cells were seeded in the upper chamber with serum‐free DMEM, while the lower chamber contained DMEM with 20% serum. After 48 h of incubation, cells on the lower surface of the membrane were fixed with paraformaldehyde and stained with 0.01% crystal violet.

### 2.11 Statistical analysis

Statistical analysis and visualization were conducted using R version 4.3.1. The Wilcoxon test was employed to assess the differences in continuous variables between two groups, while the Fisher's exact test was used to examine differences in categorical variables between the two groups. Kaplan–Meier survival analysis was performed and assessed using the log‐rank test. The correlation between continuous variables was evaluated using the Spearman or Pearson correlation method.

## 3 RESULTS

### 3.1 Overview of the genetic landscape of SLCs and establishment of a 7‐SLCs based risk signature

**FIGURE 2:**
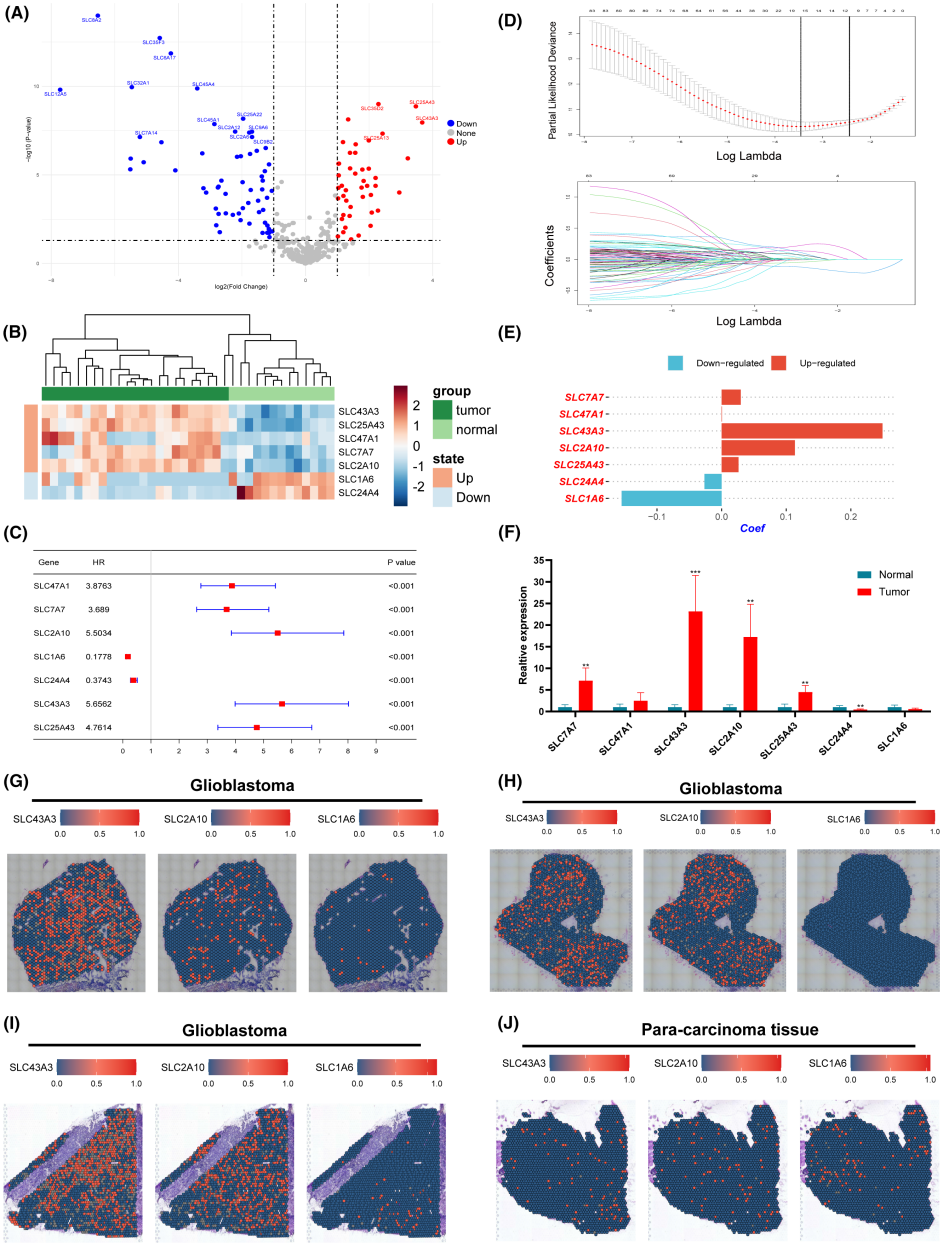
Selection and Validation of 7 Core SLCs. (A) Differential expression of SLC genes between tumour and normal tissues identified using the limma package. (B) Expression profiles of the seven core SLCs in tumour and normal tissues. (C) Univariate Cox hazard analysis reveals the prognostic value of the seven core SLCs in the TCGA pan‐glioma cohort. (D) Core SLCs selected through the lasso‐COX algorithm. (E) Regression coefficient weights corresponding to the seven core SLCs. (F) qPCR analysis of the expression levels of the seven core SLCs in tumour and adjacent normal brain tissues. (G–J) Spatial transcriptome data validate the differential expression of the top three SLCs with the highest coefficients in glioblastoma and adjacent non‐tumour tissues. *p < 0.05, **p < 0.01, ***p < 0.001. Error bars indicate the mean ± SD.

Divergent profiles of copy number alterations, somatic mutations and methylation status of SLCs were observed when analysing their genetic landscape variations in lower‐grade glioma and glioblastoma (Figure S1A–H). Next, we conducted a differential expression analysis of SLC genes between normal brain tissue and tumour tissue. The results indicate that, in comparison with normal brain tissue, the expression of several SLCs is significantly altered in tumour tissue (Figure 2A). Furthermore, employing univariate Cox analysis, we identified SLCs with prognostic value in TCGA pan‐glioma cohort. Subsequently, we subjected both differentially expressed and prognostically significant SLCs to LASSO‐COX analysis, resulting in the identification of 7 core SLCs (Figure 2B–E). Among the final 7 SLC genes we selected, SLC43A3, SLC2A10, SLC25A43, SLC7A7 and SLC47A1 exhibited upregulated expression in tumour tissue with HR greater than 1, while SLC1A6 and SLC24A4 displayed downregulated expression with HR values less than 1 (Figure 2B,C).

To validate our findings, we performed qPCR to confirm the expression levels of the seven selected SLCs in gliomas compared to adjacent non‐tumorous tissues. Five pairs of glioma tissues and their matched adjacent non‐tumorous tissues were examined. The results were consistent with our initial analysis (Figure 2F). Additionally, we utilized spatial transcriptomic data to analyse the expression of the top 3 SLCs (SLC43A3, SLC2A10 and SLC1A6) with the highest risk coefficients among the 7‐SLC signature. The results demonstrated significantly higher expression of SLC43A3 and SLC2A10 in tumour slices compared to adjacent non‐cancerous tissue slice, with almost no detectable expression of SLC1A6 in the tumour slices (Figure 2G–J). Finally, the risk score was calculated according to the following formula: $\text{Risk score}=\sum_{n=1}^{7}\left(\exp_{n}*{\text{coef}}_{n}\right)$.

### 3.2 Validation of prognostic value of a 7‐SLCs based risk signature in internal and external datasets

**FIGURE 3:**
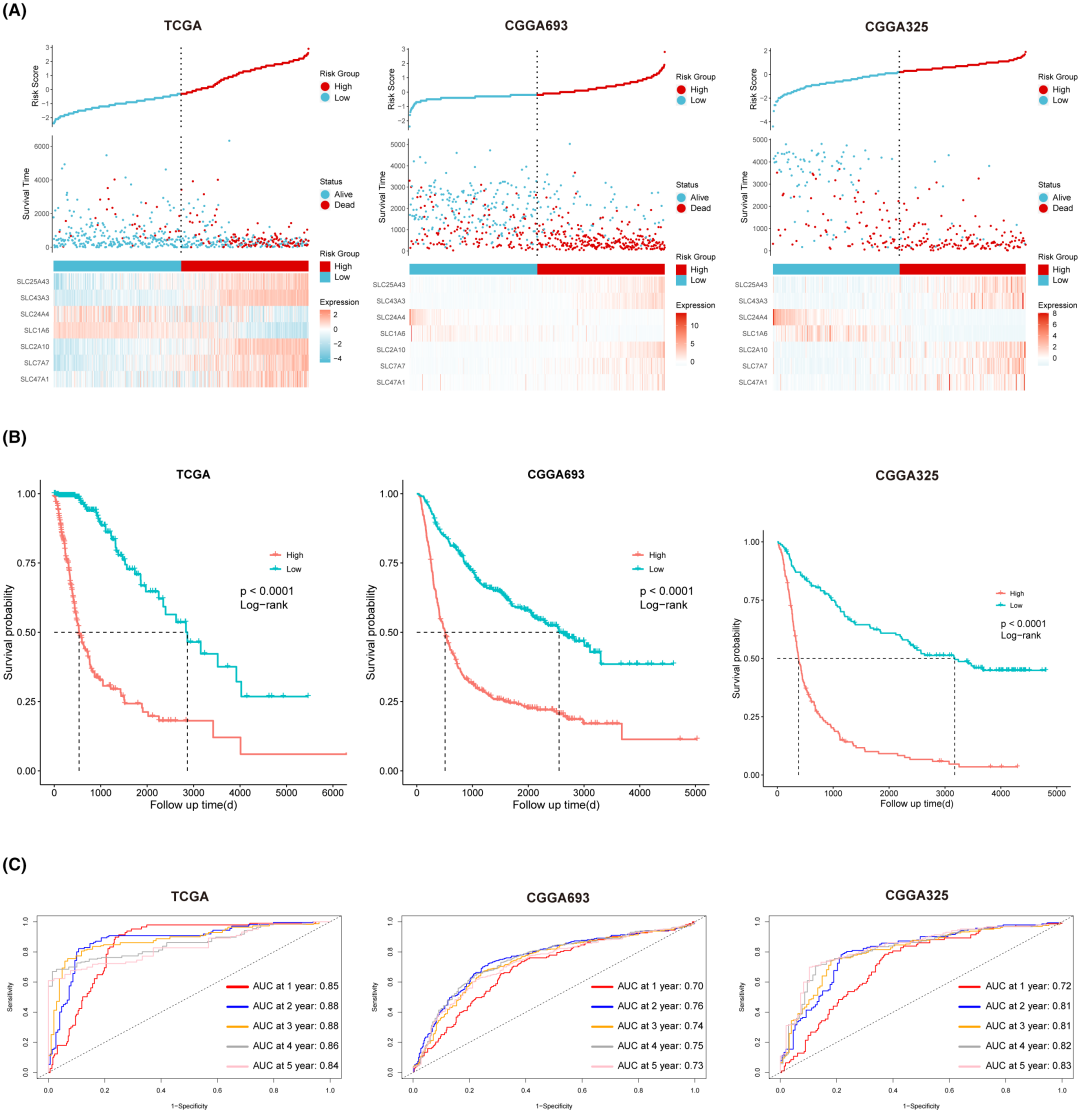
Establishment and Validation of the Risk Score Based on 7‐SLCs. (A) Distribution of risk scores, survival status and the expression profiles of the seven core SLCs in three datasets (from left to right: TCGA pan‐glioma, CGGA693, CGGA325). (B) Kaplan–Meier survival analysis reveals significantly poorer prognosis in patients belonging to the high‐risk group. (C) Time‐dependent ROC curve analysis demonstrates the good accuracy and specificity of the 7‐SLCs risk score in predicting prognosis within 1–5 years.

Using the aforementioned formula, we calculated risk scores for patients in three different cohorts, namely TCGA pan‐glioma, CGGA693 and CGGA325. Patients were divided into high‐risk and low‐risk groups according to their median risk scores. The results revealed that, across all three cohorts, patients in the high‐risk group exhibited a higher mortality rate compared to those in the low‐risk group (Figure 3A). Furthermore, Kaplan–Meier survival curve analysis indicated that, in all three independent cohorts, patients in the low‐risk group had significantly higher overall survival rates compared to the high‐risk group (Figure 3B). Finally, we conducted time‐dependent ROC curve analysis on the three independent datasets. The results showed that, in all three datasets, the area under the curve (AUC) for 1–5 years was greater than or equal to 0.70, confirming the excellent discriminative ability of the risk score (Figure 3C).

### 3.3 Establishing a nomogram and assessing its performance in internal and external datasets

**FIGURE 4:**
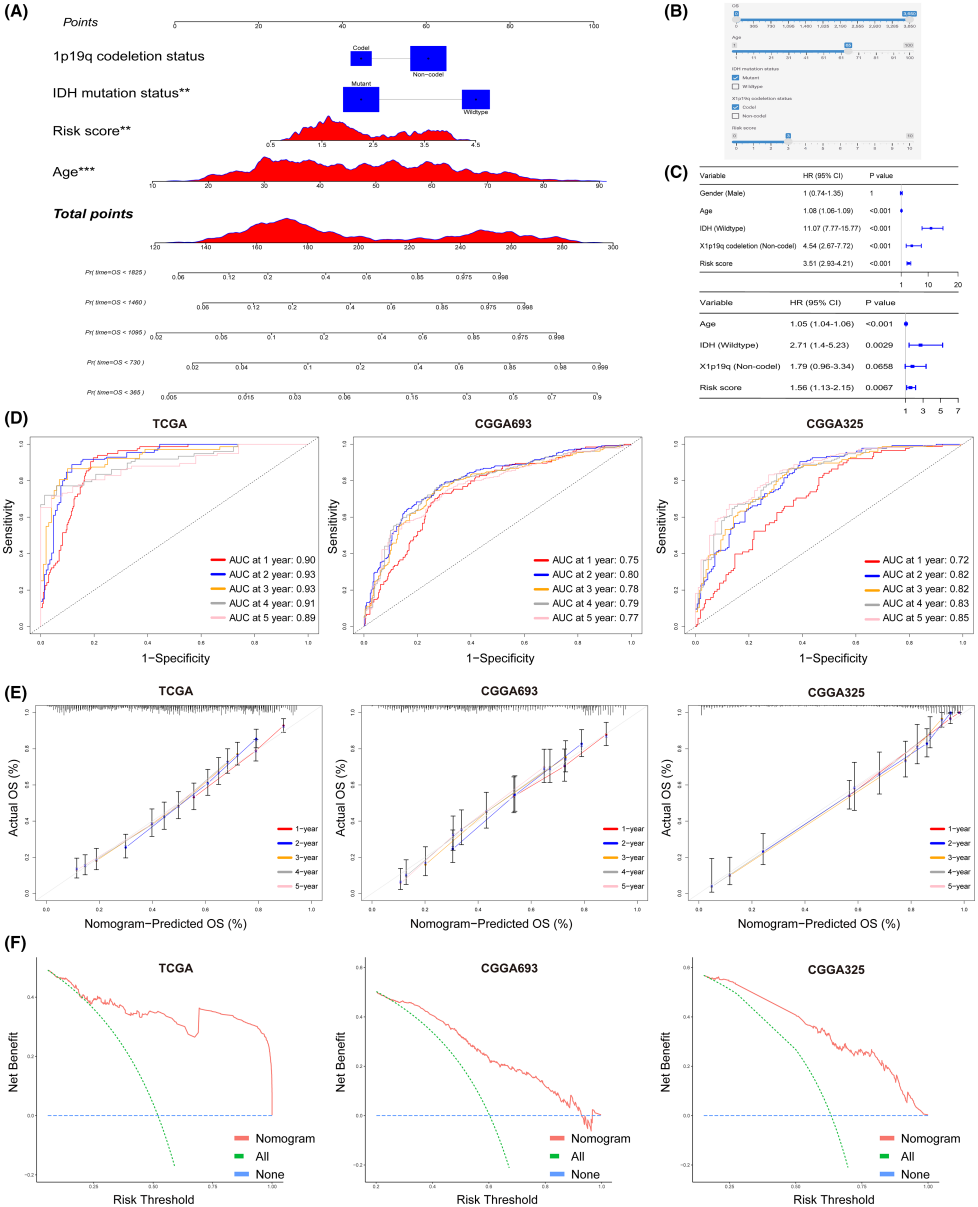
Establishment and Validation of the Nomogram. (A) The nomogram is developed by combining the risk score with other clinical and pathological features. (B) An interactive version of the nomogram is available on the website at https://net‐visualization.shinyapps.io/Nomogram/. (C) Univariate Cox and multivariate Cox analyses determine the independent prognostic value of the risk score. (D) Time‐dependent ROC curve analysis confirms the good specificity and accuracy of the nomogram. (E) Calibration curves validate the consistency between the nomogram's predicted probability and observed probability. (F) Decision curve analysis (DCA) demonstrates the benefits of the nomogram.

In conjunction with other clinicopathological features, we constructed a nomogram based on the TCGA pan‐glioma cohort to predict the prognosis of glioma patients (Figure 4A). For practical application, we developed a web page using Shinyapp platform, allowing users to interactively utilize the nomogram for predicting patient prognosis via a user‐friendly interface (Figure 4B). The web page is currently located at: https://net‐visualization.shinyapps.io/Nomogram/. Univariate and multivariate Cox analyses confirmed the independence of the risk score from other common clinicopathological characteristics (Figure 4C).

Subsequently, we assessed the performance of the nomogram in three independent datasets. Time‐dependent ROC curve analysis indicated that in the training set, TCGA pan‐glioma, the AUC for 1 to 5 years was close to 0.9, and in the external test sets, CGGA693 and CGGA325, the model's AUC for 1 to 5 years was also greater than 0.7. This suggests that the nomogram exhibits excellent discriminatory ability (Figure 4D). Similarly, the calibration curves for the three independent datasets also indicated that the model exhibited good accuracy in predicting probabilities for 1 to 5 years (Figure 4E). DCA further demonstrated that the nomogram could benefit patients (Figure 4F).

### 3.4 Differences in functional pathways and immune microenvironment between high‐ and low‐risk groups

First, we conducted differential gene expression analysis between the high‐risk and low‐risk score groups. Subsequently, we intersected the top 500 upregulated and downregulated genes from each of the three datasets and performed functional enrichment analysis on the obtained common upregulated or downregulated intersecting genes (Figure S2A,B). We found that the commonly upregulated genes were significantly enriched in immune regulation, extracellular matrix remodelling and metabolism‐related pathways in three different databases (GO, KEGG, Reactome) (Figure S2C–E). In contrast, the commonly downregulated genes were primarily enriched in normal neural system function‐related entries (Figure S2F–H). This suggests that the high‐risk group experiences alterations in immune, metabolic and tumour microenvironment relative to the low‐risk score group.

**FIGURE 5:**
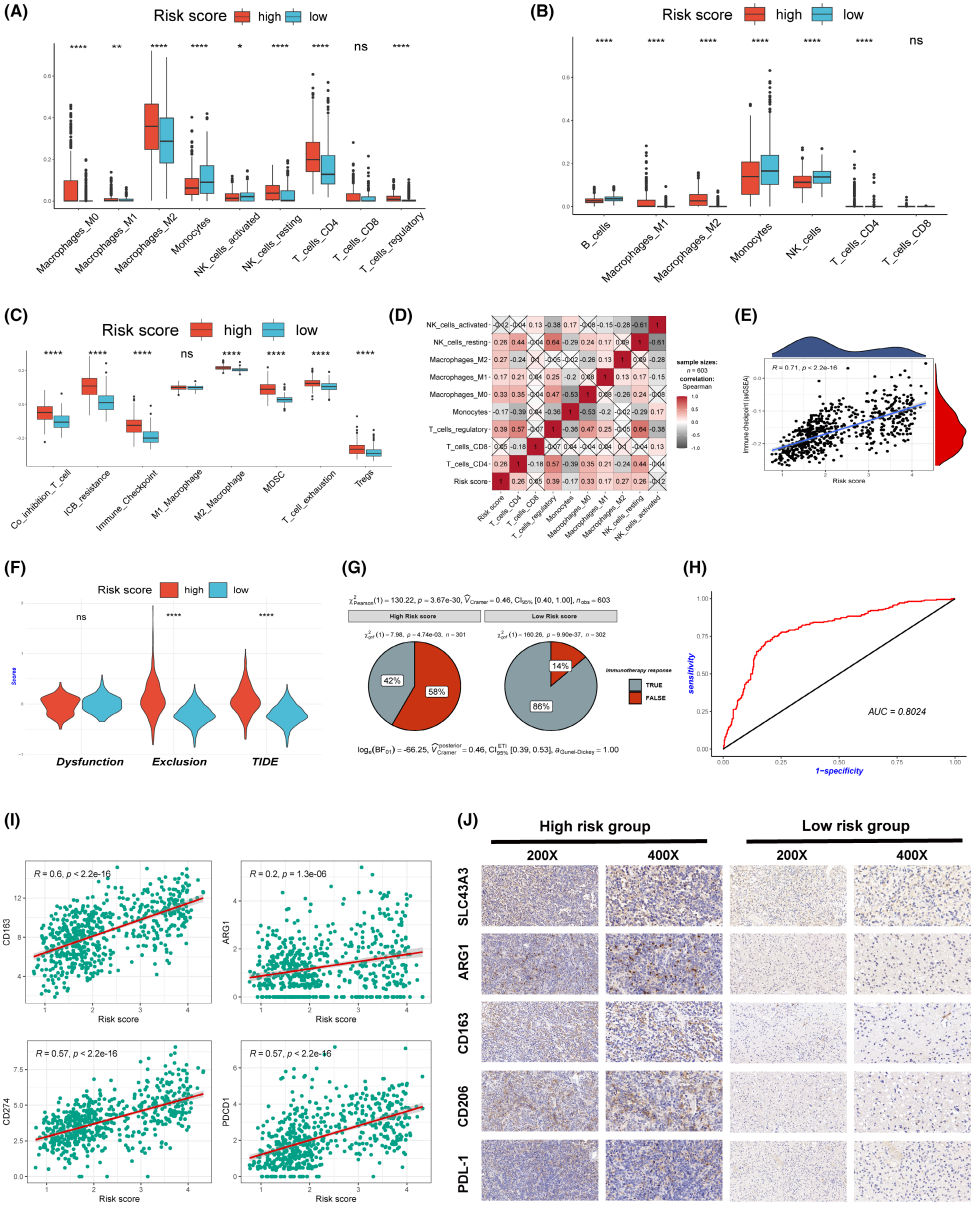
Different immune microenvironments exist in the high‐ and low‐risk groups. Three distinct algorithms, including CIBERSORT (A), quanTIseq (B) and ssGSEA (C), confirmed the presence of a more immunosuppressive microenvironment in the high‐risk group. (D) The correlation between risk scores and the proportions of different immune cell infiltrations. (E) The correlation between risk scores and immune checkpoint ssGSEA scores. (F) The TIDE algorithm predicts immune‐related scores in high‐ and low‐risk groups. (G) The TIDE algorithm predicts the response of high‐ and low‐risk groups to immunotherapy. (H) ROC curve analysis indicates that risk scores can differentiate potential responses to immunotherapy. (I) Analysis of the correlation between immune scores and M2 macrophage markers, PD‐1 and PD‐L1. (J) Immunohistochemistry confirms a higher presence of M2 macrophages and PD‐1 in the high‐risk group.

To further investigate the differences in the immune microenvironment between the two groups, we performed immune infiltration analysis on the high‐risk and low‐risk score groups using three different algorithms (CIBERSORT, quanTIseq and ssGSEA). The results revealed that the high‐risk group had a higher proportion of immune‐suppressive cell infiltrates, such as regulatory T cells and M2‐type macrophages (Figure 5A–D). Additionally, ssGSEA analysis indicated that the high‐risk group had higher immune checkpoint scores, T‐cell exhaustion scores and immune checkpoint inhibitor resistance scores (Figure 5C). We found a significant positive correlation between the risk score and the infiltration proportion of M2‐type macrophages as well as the immune checkpoint score (Figure 5D,E). Based on these findings, we used the TIDE algorithm to predict the potential response to immunotherapy in the high‐risk and low‐risk groups. The results indicated that patients in the high‐risk group had significantly lower response rates to immunotherapy, with higher exclusion and TIDE scores (Figure 5F,G). ROC curve analysis confirmed that the risk score had good discriminative ability for predicting the response to immunotherapy (Figure 5H). We further validated the discriminative ability of the risk score in three external real‐world immunotherapy cohorts (Figure S3A–C). Besides, we observed a significant positive correlation between risk scores and the expression of M2‐type macrophage markers (CD163, ARG1) and classical immune checkpoint molecules (PD‐L1, PD‐1) (Figure 5I). This evidence supports the notion that a more immunosuppressive tumour microenvironment may exist in the high‐risk group.

To validate our findings, we collected 20 surgical specimens from glioma patients and stratified them into high‐risk and low‐risk groups based on the distinct expression patterns of the seven SLCs examined by qPCR. Immunohistochemistry assays confirmed higher expression levels of ARG1, CD163, CD206 and PD‐L1 in the high‐risk group (Figure 5J).

### 3.5 The association of 7‐SLCs based risk score with metabolic status and drug sensitivity

**FIGURE 6:**
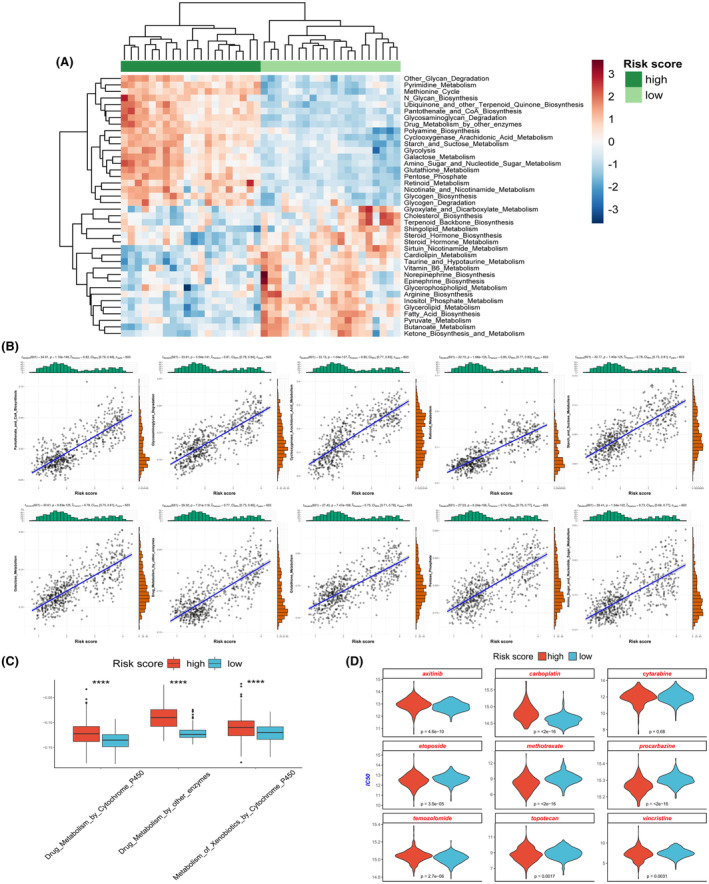
Different metabolic pathway activities and varying sensitivities to chemotherapy drugs exist in the high‐ and low‐risk groups. (A) Distinct metabolic pathway activation states in the high‐ and low‐risk groups. (B) Risk scores exhibit a significant correlation with the ssGSEA scores of various metabolic pathways. (C) Differential activity of drug metabolism‐related metabolic pathways is observed in the high‐ and low‐risk groups. (D) Prediction of the sensitivity of high‐ and low‐risk groups to chemotherapy drugs.

We utilized the ssGSEA algorithm to assess the metabolic pathway activities in both the high‐risk and low‐risk groups. The results indicated strikingly different metabolic states between these groups (Figure 6A). The high‐risk score group exhibited higher activity in metabolic pathways such as fructose metabolism, glycolysis, pentose phosphate pathway and drug metabolism (Figure 6A,C). Correlation analysis further confirmed a significant positive correlation between the risk score and the activity of these metabolic pathways (Figure 6B). We additionally evaluated the sensitivity of patients in both high‐risk and low‐risk groups to commonly employed chemotherapy drugs for glioma. The results indicated that patients in different groups exhibited varying sensitivities to different drugs. Compared to the low‐risk group, the high‐risk group showed significantly higher resistance to drugs such as temozolomide, carboplatin and axitinib, while they exhibited higher sensitivity to drugs like etoposide, topotecan, vincristine, methotrexate and procarbazine (Figure 6D).

### 3.6 Heterogeneity of 7‐SLCs based risk score at single‐cell resolution

**FIGURE 7:**
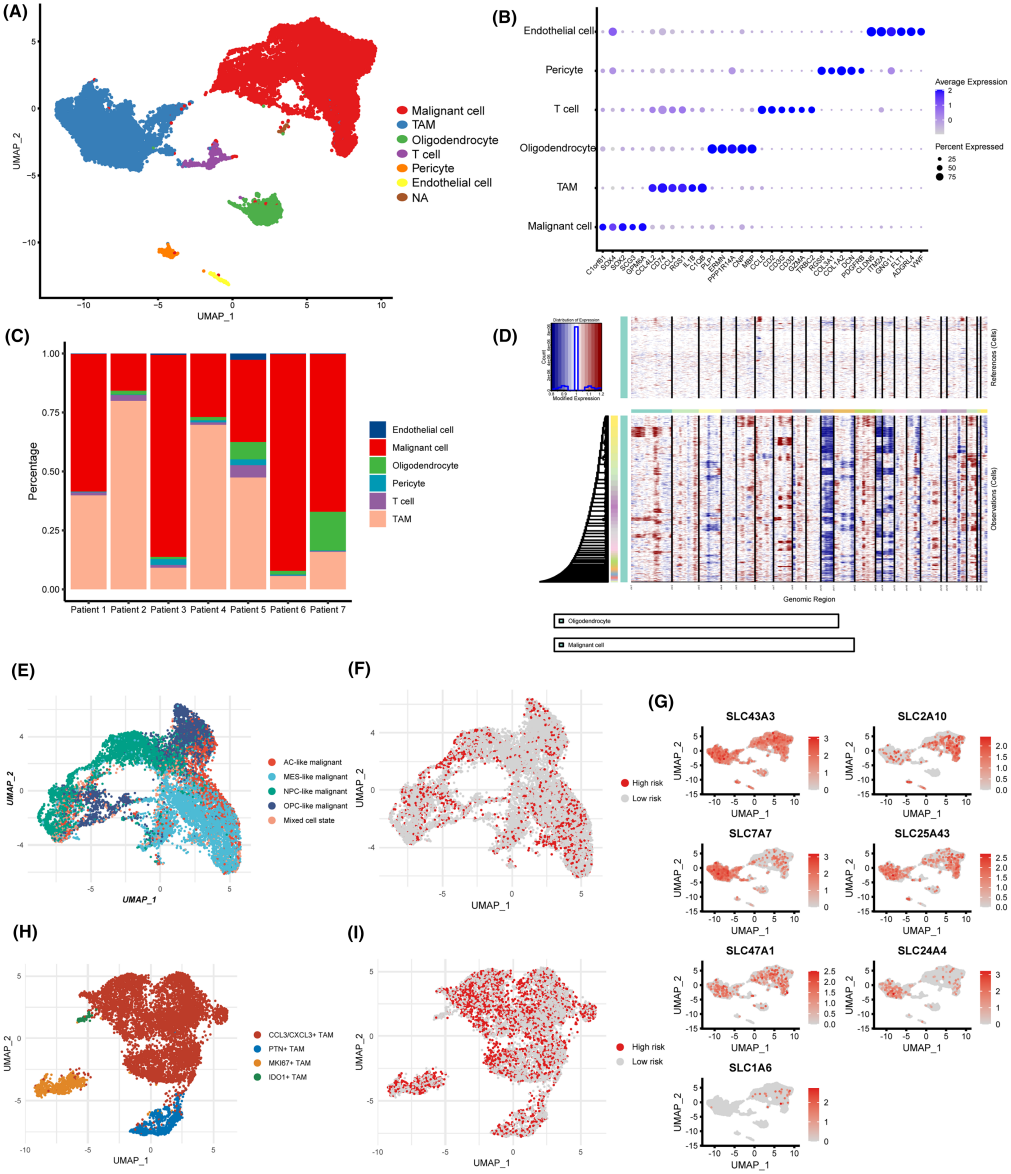
Expression patterns of the 7 core SLCs at the single‐cell resolution. (A) Clustering and annotation of single‐cell sequencing data from glioblastoma. (B) Marker genes expressed in different cell clusters. (C) Cell composition in different patient samples. (D) Copy number variation analysis in malignant cells. (E) Cell state annotation in malignant cells. (F) Determination of different SLC expression patterns in malignant cells. (G) Expression profiles of the seven core SLCs in different cell subpopulations. (H) Further annotation of TAMs subpopulations. (I) Determination of different SLC expression patterns in TAMs.

We performed standard pipeline analysis on the single‐cell sequencing dataset GSE135045. Due to observed batch effects in the data, we applied the Harmony algorithm to remove batch effects. Subsequently, based on specific markers expressed in different cell subtypes, 23,208 cells were annotated as malignant cells, TAMs, oligodendrocytes, pericytes, endothelial cells and T cells, respectively (Figure 7A,B). We observed variations in cell composition originating from different patient samples. However, tumour cells and TAMs constituted the majority in each patient sample (Figure 7C). Subsequently, inferCNV analysis confirmed that tumour cells exhibited significant CNV events, such as chromosome 7 amplification and chromosome 10 deletion, in comparison with oligodendrocytes (Figure 7D). This further substantiates the accuracy of our annotation for tumour cells.

Subsequently, we categorized tumour cells into five distinct cellular states based on the differential expression patterns of specific marker genes. These states are referred to as AC‐like, MES‐like, OPC‐like, NPC‐like and a mixed‐cell state (Figure 7E). According to the previously established risk score formula, malignant tumour cells were classified into high‐risk tumour cells and low‐risk tumour cells based on the distinct expression patterns of the seven SLC genes (Figure 7F). We further examined the expression of the seven SLC genes that constitute the risk score in different cellular subpopulations. The results indicate that SLC43A3, SLC25A43, SLC2A10 and SLC47A1 show sporadic expression in both tumour cells and TAMs. In contrast, SLC7A7 and SLC24A4 exhibit a more widespread expression in TAMs, while SLC1A6 was detected in only a small fraction of tumour cells (Figure 7G). We observed that high‐risk score malignant cells are scattered across various cellular states, indicating that the cellular states of high‐risk score cells are heterogeneous and not confined to a uniform state (Figure 7E,F and Figure S4K). Similarly, we reclassified TAMs into subgroups and categorized them into high‐risk or low‐risk groups based on the distinctive expression patterns of seven specific SLCs (Figure 7H,I). Since conventional binary classification of TAMs into M1 and M2 types is challenging,^38^ we subdivided them into four distinct subgroups based on unique transcriptional patterns: CCL3/CXCL3+ TAMs, PTN+ TAMs, MKI67+ TAMs and IDO1+ TAMs (Figure 7H; Figure S4A–J). Similar to the distribution of malignant cells in the high‐risk group, we also observed that TAMs in the high‐risk group consist of cells with various transcriptional characteristics (Figure 7H,I; Figure S4L).

### 3.7 Differential transcriptome regulation and metabolic states in distinct 7‐SLC expression patterns within malignant cells

**FIGURE 8:**
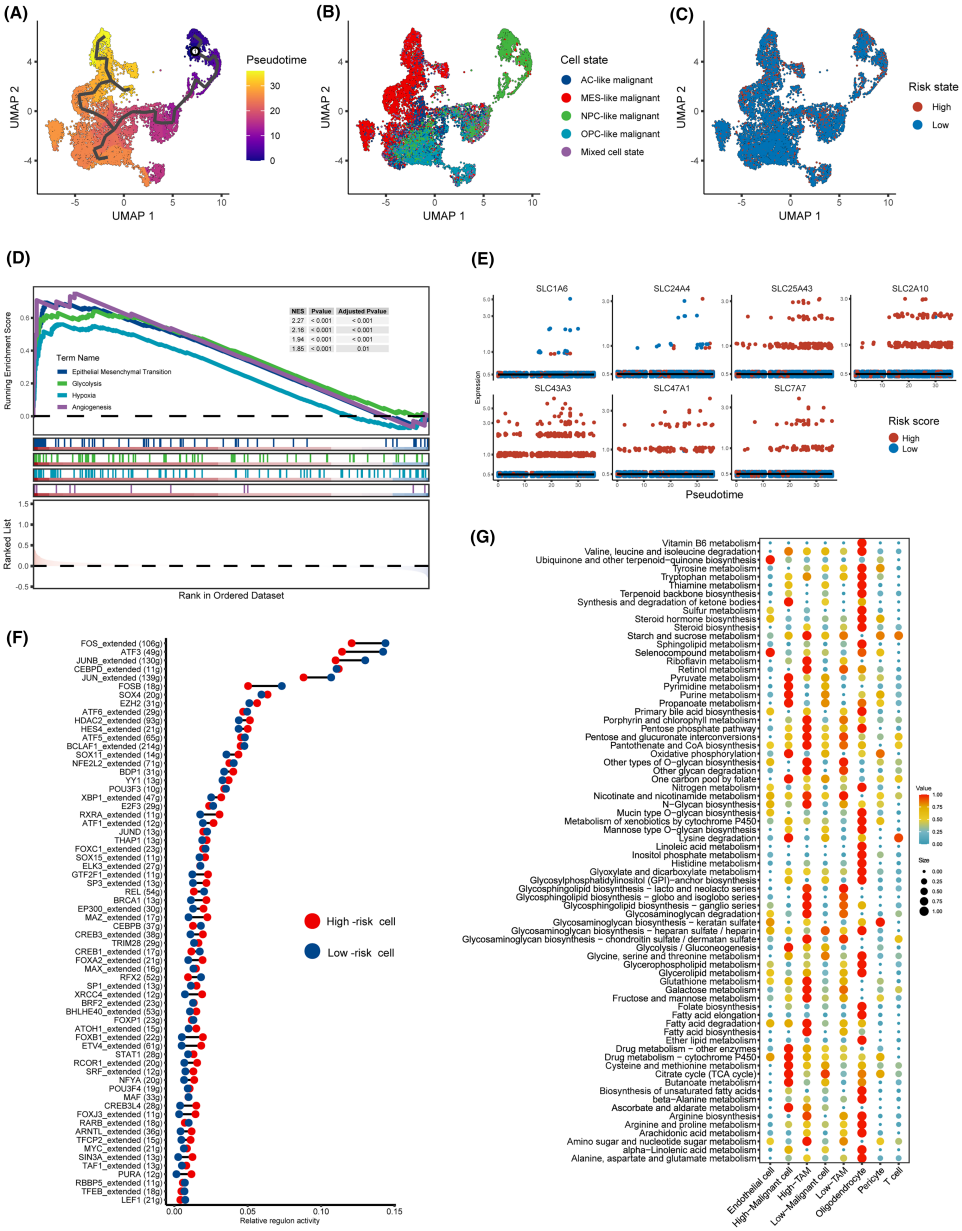
The inherent differences between cells with two different SLC expression patterns (high‐risk and low‐risk cells). (A) Pseudo‐temporal analysis of malignant cells. (B) The position of malignant cells in the differentiation trajectory across different cell states. (C) The position of high‐ and low‐risk malignant cells in the differentiation trajectory. (D) Enrichment analysis results of GSEA in high‐risk malignant cells. (E) Expression profiles of the seven core SLCs along the differentiation trajectory. (F) Differences in the activity of transcriptional regulatory factors in high‐ and low‐risk malignant cells. (G) Differences in the activity of metabolic pathways in high‐ and low‐risk malignant cells and TAMs.

Based on the distinct expression patterns of seven SLC genes in malignant cells and TAMs, we categorized these cells into high‐risk and low‐risk cell groups. Using the Monocle3 algorithm, we conducted pseudotime analysis on malignant cells. The results indicate that NPC‐like malignant cells are situated at the initiation stage of differentiation, while MES‐like malignant cells are located at the terminal stage of differentiation (Figure 8A,B). This suggests the presence of a dynamic transition process within glioblastomas, potentially involving the transformation of NPC‐like cells into MES‐like cells and various intermediate states. Furthermore, we observed the presence of highly scored malignant cells throughout the entire differentiation trajectory (Figure 8C). GSEA analysis revealed a significant enrichment of hypoxia, angiogenesis, glycolysis and epithelial‐mesenchymal transition pathways in high‐risk cells (Figure 8D). While simultaneously analysing the expression profiles of seven core SLC genes along the differentiation trajectory, no significant changes in SLC expression patterns were observed (Figure 8E).

Subsequently, we employed the SCENIC algorithm and the scMetabolism algorithm to assess differences in transcriptional regulatory networks and metabolic states between high‐ and low‐risk‐score groups of malignant cells. We observed differences in the activity of multiple regulons between the two cell groups, such as ATF3 and FOSB (Figure 8F). This suggests the presence of intrinsic, non‐uniform transcriptional regulatory patterns in these two groups of cells. Furthermore, inference of metabolic pathway activity unveiled distinct metabolic patterns in the two cell groups. We found that high‐risk malignant cells exhibited heightened activity in purine metabolism and glycolysis, while high‐risk TAMs displayed increased activity in arginine synthesis and fatty acid metabolism (Figure 8G). Interestingly, SLC43A3, the most heavily weighted among the seven SLC genes, has previously been reported to be associated with purine and fatty acid metabolism.^39^, ^40^ These results further confirmed that our classification based on the 7‐SLC expression pattern can distinguish between two intrinsically different sets of cell subpopulations.

### 3.8 Unique cellular communication patterns in high‐risk scoring malignant cells and TAMs

**FIGURE 9:**
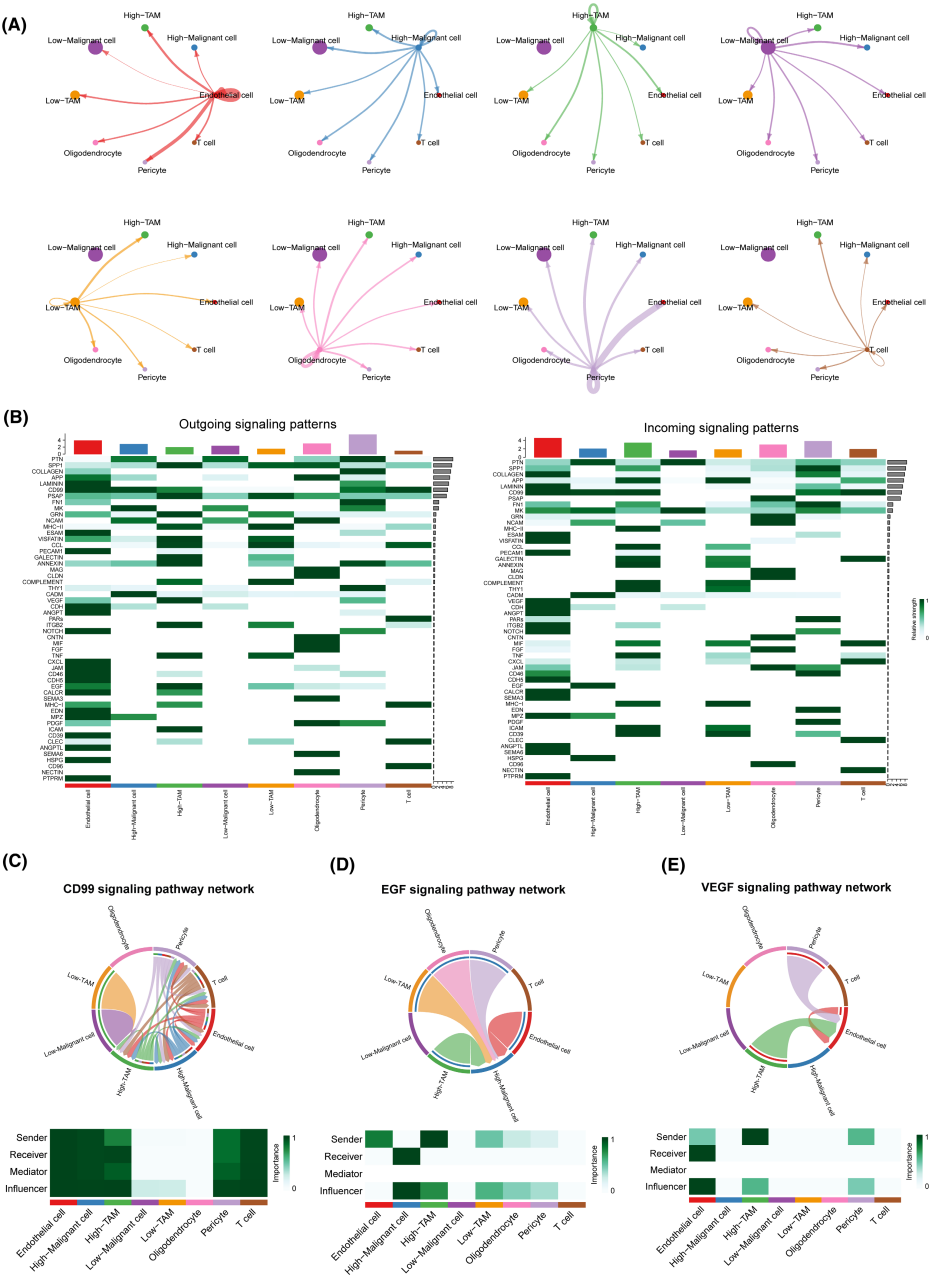
Cells with distinct SLC expression patterns exhibit unique cell–cell communication. (A) Complex interactions exist among different cell subpopulations. (B) Signals emitted and received by different cell subpopulations. (C–E) The roles of different cell subpopulations in the CD99, EGF and VEGF signalling networks.

To explore whether high‐risk cells and low‐risk cells differ in their interactions with other components in the microenvironment, we conducted cellular communication analysis using the CellChat algorithm. The results revealed unique cell communication patterns within the high‐risk cell group (Figure 9A,B). In comparison with the low‐risk group, malignant cells in the high‐risk group exhibited significantly stronger activity in the HSPG, EGF, CD99 and MPZ signalling pathways, while TAMs in the high‐risk group displayed heightened activity in the VEGF and ANNEXIN signalling pathway networks (Figure 9B–E).

We simultaneously analysed specific ligand–receptor pairs from each signalling pathway network. The HSPG signalling, primarily mediated by HSPG2‐DAG1, was predominantly emitted by endothelial cells and received by malignant cells in the high‐risk group (Figure 9B; Figure S5B,C). The EGF signalling, mainly mediated by HBEGF‐EGFR, was emitted by TAMs, endothelial cells, pericytes and oligodendrocytes, and received by high‐risk malignant cells (Figure 9D; Figure S5B,C). The MPZ signalling network, mediated by several ligand–receptor pairs, was primarily concentrated within endothelial cells and malignant cells in the high‐risk group (Figure 9B). Conversely, the CD99 signalling network, mediated by multiple ligand–receptor pairs, exhibited intricate network activity, involving nearly all cell types except oligodendrocytes (Figure 9C). The VEGF signalling network, mediated by VEGFA‐VEGFR1/VEGFR2/VEGFR1R2, was primarily concentrated between high‐risk scoring TAMs, pericyte and endothelial cells, suggesting that high‐risk scoring TAMs may promote angiogenesis in glioblastoma (Figure 9E; Figure S5D,E).

**FIGURE 10:**
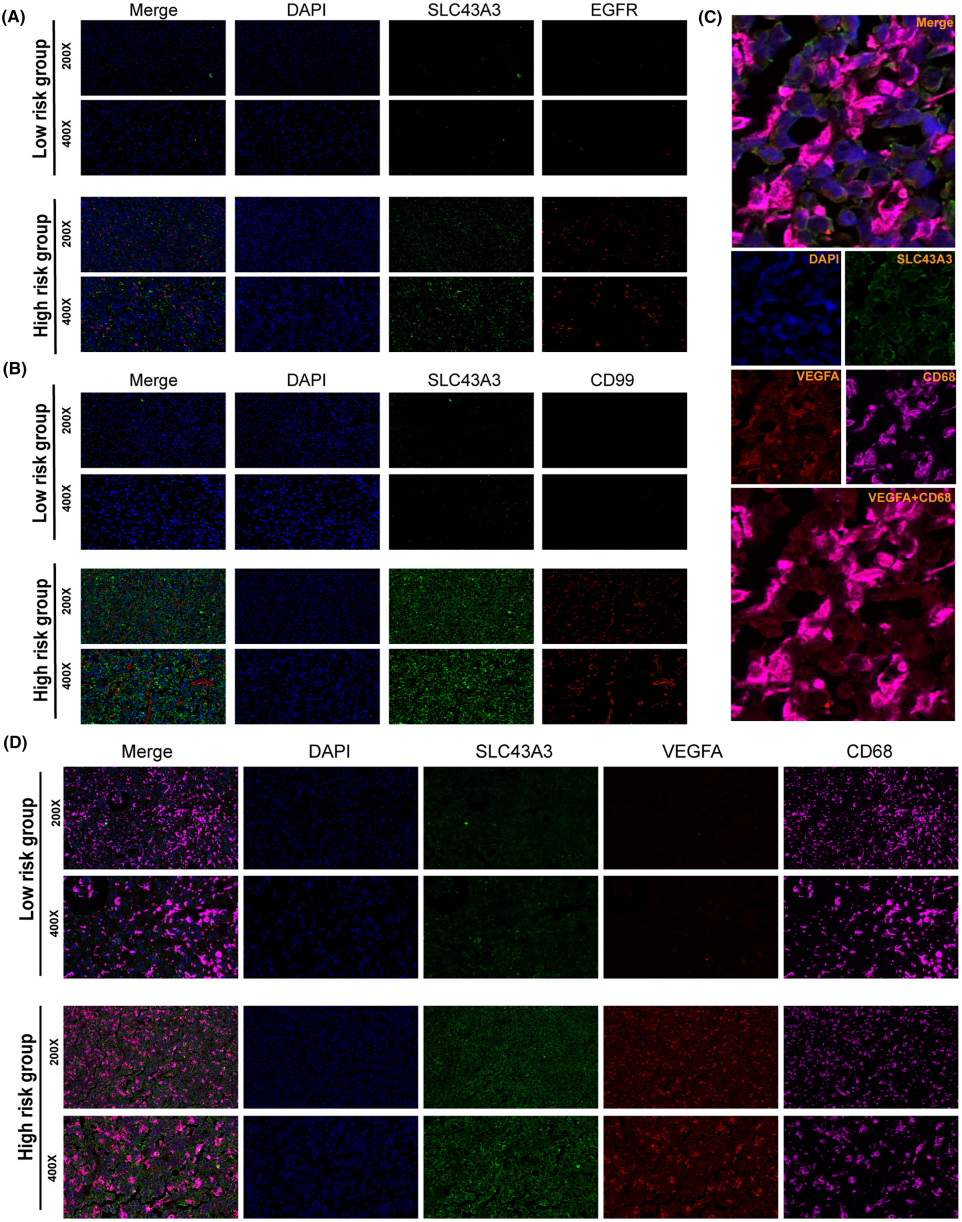
Immunofluorescence validation of key gene expression. (A) Samples from the high‐risk group showed a higher level of EGFR expression. (B) Samples from the high‐risk group exhibited greater CD99 expression. (C) Co‐localization staining of CD68, SLC43A3 and VEGFA revealed the presence of a higher number of pro‐angiogenic TAM subpopulations in the high‐risk group. (D) Samples from the high‐risk group showed higher VEGFA expression levels.

To validate our analysis, immunofluorescence experiments were conducted. The results demonstrated that, in comparison with tissues with low‐risk scores, tissues with high‐risk scores exhibited increased expression of SLC43A3, CD99, EGFR and VEGFA (Figure 10A–D). Similar conclusions were reached through immunohistochemical staining of the high‐risk and low‐risk groups (Figure S5A). In the high‐risk group, we performed co‐localization analysis of TAMs using CD68, VEGFA and SLC43A3 (SLC43A3 being the highest weighted SLC in the expression pattern determination). The results revealed that TAMs secreting VEGFA exhibited concomitant high expression of SLC43A3. This suggests that a specific expression pattern of SLCs may be intrinsically associated with the subpopulation of TAMs that secrete VEGFA (Figure 10C,D).

### 3.9 The distinctive contribution of SLC43A3 to risk scoring and its potential functions in glioma

**FIGURE 11:**
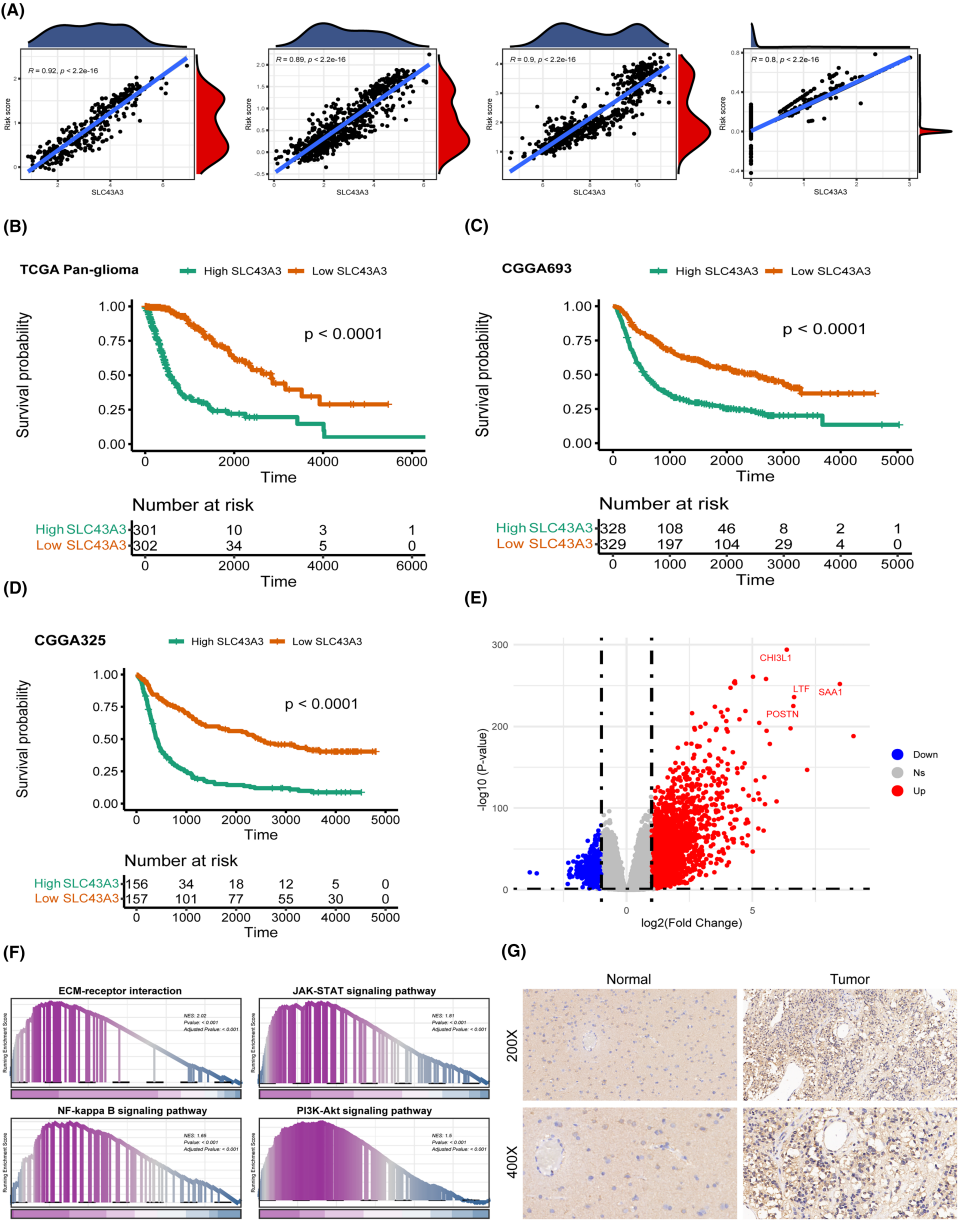
Potential role of the orphan protein SLC43A3 in glioma. (A) SLC43A3 expression and risk scores showed a significant positive correlation. (B–D) Patients with high expression of SLC43A3 have significantly worse prognosis (divided by median expression of SLC43A3). (E) Differential genes between the high and low SLC43A3 expression groups. (F) Results of GSEA enrichment analysis in the highly expressed SLC43A3 group. (G) Immunohistochemistry confirmed the high expression of SLC43A3 in glioma tissues compared to normal brain tissues.

Given that SLC43A3 holds the highest weight in our scoring model, we conducted a correlation analysis between SLC43A3 and risk scoring across four distinct data sources. The results consistently demonstrate a significant positive correlation between SLC43A3 and risk scores in various datasets (Figure 11A). Additionally, Kaplan–Meier survival analyses conducted on three distinct datasets, including TCGA pan‐glioma, CGGA325 and CGGA693, further indicate that high expression of SLC43A3 is an adverse prognostic factor (Figure 11B–D). Furthermore, we conducted differential analysis (Figure 11E) and functional enrichment analysis on groups characterized by high and low SLC43A3 expression. The results revealed a significant enrichment of signalling pathways associated with tumour progression, such as PI3K‐Akt and JAK–STAT, in the high SLC43A3 expression group (Figure 11F). Immunohistochemical staining of glioma tissue and normal brain tissue also confirmed the significant overexpression of SLC43A3 in gliomas (Figure 11G).

**FIGURE 12:**
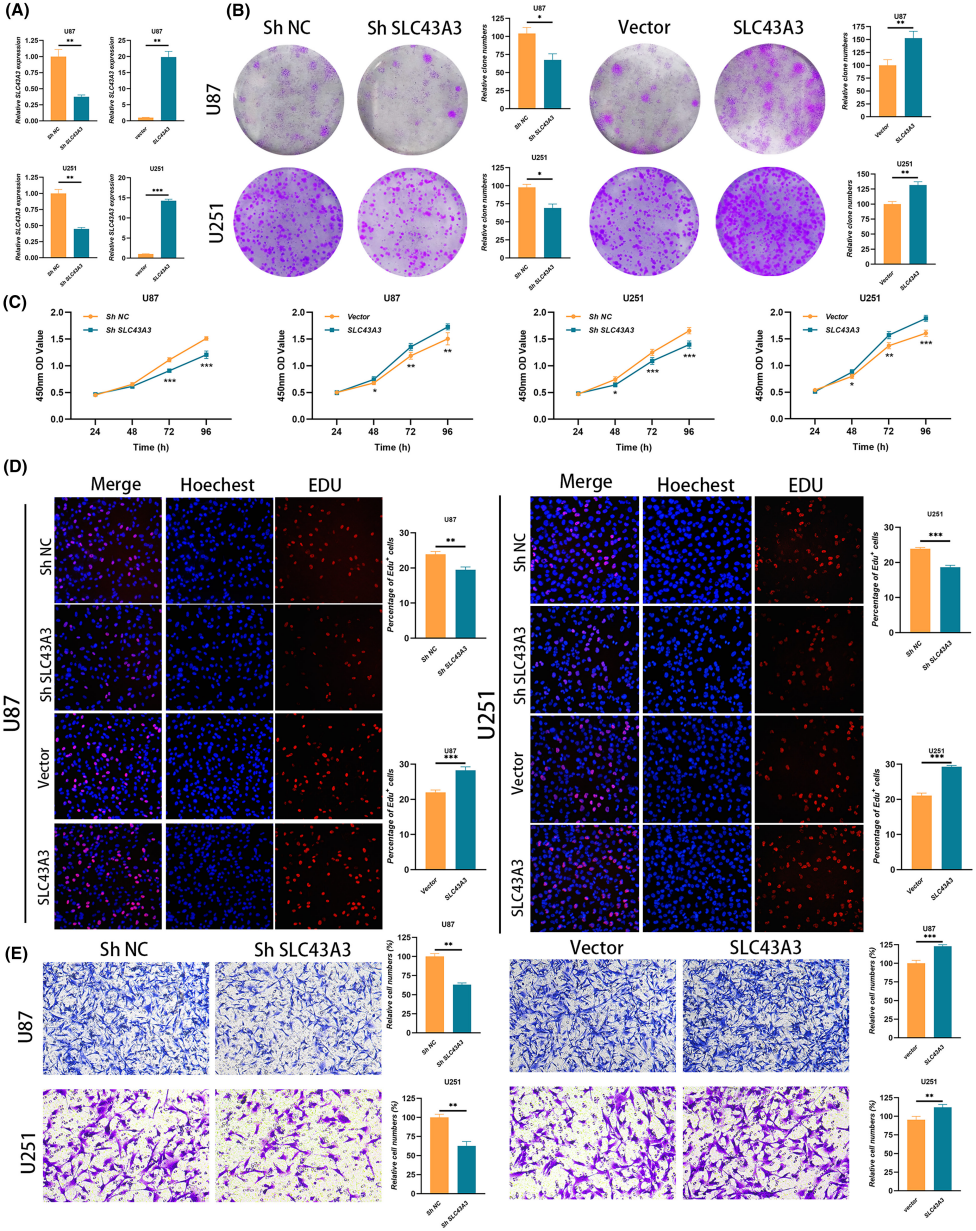
SLC43A3 promoted the proliferation and migration of glioma cells in vitro. (A) Expression levels of SLC43A3 were manipulated in U87 and U251 cell lines, with upregulation or downregulation confirmed through qPCR. (B) Cell colony formation assays were conducted after modulating the expression levels of SLC43A3 in U87 and U251 cells. (C) CCK8 experiments were performed after adjusting the expression levels of SLC43A3 in U87 and U251 cells. (D) Edu experiments were carried out after adjusting the expression levels of SLC43A3 in U87 and U251 cells. (E) Transwell experiments were conducted after adjusting the expression levels of SLC43A3 in U87 and U251 cells.

Subsequently, we manipulated the expression levels of SLC43A3 in U87 and U251 cell lines, inducing either upregulation or downregulation (Figure 12A). Results from cell colony formation assays (Figure 12B) and CCK8 experiments (Figure 12C) demonstrated a significant reduction in the growth potential of U87 and U251 cells upon downregulation of SLC43A3 expression. Edu experiments provided additional evidence, confirming a distinct suppression in the proliferation of tumour cells after SLC43A3 downregulation (Figure 12D). The migratory capacity of tumour cells was markedly hindered after the downregulation of SLC43A3 (Figure 12E). Conversely, overexpression of SLC43A3 resulted in effects opposite to these phenomena.

## 4 DISCUSSION

Solute carrier proteins (SLCs), as transmembrane transporters for various endogenous and exogenous substances, have seen over 450 members identified to date.^41^ They have been extensively associated with several hallmarks of cancer, including metabolic reprogramming, proliferation, migration, angiogenesis and immune regulation.^15^, ^16^, ^41^ Many SLCs that undergo changes in expression to adapt to alterations in tumour metabolism and SLCs related to chemotherapy drug metabolism have long been considered potential targets for cancer therapy.^15^ Recently, in light of the deepening understanding of the tumour microenvironment, the role of SLCs, which facilitate the transport of various substances between cells or between cells and the extracellular matrix, has garnered widespread attention. The tumour microenvironment is primarily composed of malignant cells, immune cells and other stromal cells, featuring complex communication networks and mutual regulatory mechanisms among various cellular components.^42^ Apart from their impact on the phenotype of malignant cells, SLCs have also been found to influence the differentiation and function of immune cells, such as the association of SLC1A5 with T‐cell activation and SLC16A3 with macrophage pro‐inflammatory phenotypes.^17^ Hence, targeting SLCs to reshape the tumour microenvironment represents a promising potential approach for cancer therapy. Nonetheless, the roles of many orphan SLCs in the formation of the tumour microenvironment remain largely unexplored.

To the best of our knowledge, this study represents the first comprehensive elucidation of the genetic landscape, prognostic value and potential role of SLCs in glioblastoma, integrating multidimensional data such as bulk/single‐cell/spatial transcriptomics. Experimental evidence has confirmed the association between the expression patterns of specific SLCs and the formation of an immune‐suppressive microenvironment, while also demonstrating that the orphan SLC protein, SLC43A3, as an oncogene, can enhance the proliferation and migration of glioblastoma cells. Initially, we conducted a genetic landscape analysis of SLCs in glioma. Subsequently, we identified SLCs that not only hold prognostic value but also display expression dysregulation in glioblastoma. Utilizing the Lasso‐Cox algorithm, we identified seven core SLCs: SLC43A3, SLC2A10, SLC25A43, SLC7A7, SLC47A1, SLC1A6 and SLC24A4. We then validated our findings using qPCR experiments and spatial transcriptome data. Among these SLCs, SLC2A10 has been reported to be associated with ferroptosis in glioblastoma,^43^ SLC47A1 has been demonstrated to mediate temozolomide resistance in glioblastoma,^44^ while the functions of the other SLCs in glioblastoma remain unclear. Next, to enhance the prediction of prognosis for glioma patients, we established a risk scoring system based on these seven core SLCs. Patients from three independent cohorts, TCGA pan‐glioma, CGGA693 and GAAA325, were subjected to risk scoring and then divided into high‐risk and low‐risk groups according to the median score. Kaplan–Meier survival analysis confirmed significantly poorer prognosis for patients in the high‐risk group across all three datasets. Time‐dependent ROC curve analysis also confirmed the accuracy and specificity of risk scoring in predicting outcomes over 1–5 years. To further improve the predictive performance of the model, we incorporated other common clinical and pathological features, such as IDH mutation status, age and 1p19q deletion status, to construct a nomogram. Time‐dependent ROC curve analysis, calibration curve analysis and decision curve analysis all validated the practical value of the nomogram. Furthermore, for clinical convenience, we developed a user‐friendly interactive web‐based dynamic nomogram.

In fact, our risk scoring system, which is based on the product of regression coefficients and expression levels, quantifies the distinct expression patterns of these seven SLCs in different patients. Given the close association of SLCs with immunity and metabolism, we further investigated the variances in immune infiltration and metabolic states among different risk groups. We observed that the high‐risk group exhibited a significantly more pronounced immune‐suppressive microenvironment, characterized by increased M2‐type macrophage infiltration, elevated expression of immune checkpoint molecules and a potential resistance to immunotherapy. To validate our analysis, we conducted immunohistochemical experiments on samples from the high‐ and low‐risk groups, confirming higher expression of M2‐type macrophage markers such as ARG1, CD163, CD206 and the immune checkpoint PDL‐1 in the high‐risk group. Additionally, the high‐risk group displayed distinct metabolic pathway activities and differential responses to chemotherapy agents, with a higher resistance to temozolomide, carboplatin and axitinib and increased sensitivity to etoposide, topotecan, vincristine, methotrexate and procarbazine. Chemotherapy agents have traditionally been the frontline treatment for glioblastoma, while immunotherapy offers new hope in glioblastoma therapy.^45^, ^46^ Therefore, grouping based on the expression patterns of different SLCs may provide valuable guidance in the selection of chemotherapy and immunotherapy for patients.

A decade ago, glioblastoma was classified into four distinct subtypes, namely proneural subtype, neural subtype, classical subtype and mesenchymal subtype, based on large‐scale bulk transcriptome sequencing.^47^, ^48^ However, with the advancement of single‐cell sequencing technologies, our understanding of the heterogeneity within the glioblastoma microenvironment has deepened. At single‐cell resolution, glioblastoma has revealed four distinct cellular states, namely the NPC‐like state, OPC‐like state, AC‐like state and MES‐like state.^33^ Inspired by this paradigm shift, we leveraged single‐cell sequencing data to evaluate the correlation between the expression patterns of specific SLCs and cellular states and functions at the single‐cell level. To begin, we performed cell type annotation on the single‐cell sequencing data, and the expression of the seven core SLCs was primarily detected in malignant cells and TAMs. Interestingly, similar to the results obtained from bulk analysis, the single‐cell data showed a higher detection of SLC43A3, SLC2A10, SLC25A43, SLC7A7 and SLC47A1, which are highly expressed in tumour tissues, relative to SLC1A6 and SLC24A4, which are lowly expressed. Given the predominant presence of malignant cells and TAMs in the composition of the glioblastoma microenvironment, we focused on analysing the heterogeneity of different SLC expression patterns within these cell populations. Malignant cells were further categorized into NPC‐like state, OPC‐like state, AC‐like state and MES‐like state based on distinct cellular conditions, whereas TAMs were annotated according to diverse transcriptional characteristics. Pseudo‐temporal analysis unveiled the dynamic transition of tumour cells from the NPC‐like state to the MES‐like state, while the corresponding SLC expression patterns did not exhibit significant changes along the differentiation trajectory. Therefore, we hypothesize that the expression patterns of SLCs may be independent of other features. Subsequently, in order to more comprehensively assess the influence of varied SLC transcription patterns on cellular functionality, we conducted further analyses on intercellular communication patterns, transcriptional regulatory modes and metabolic activities within the high‐ and low‐risk groups. Interestingly, high‐risk malignant cells exhibited heightened CD99 and EGF signal pathway network activities, while high‐risk TAMs displayed increased VEGF signal pathway network activities. CD99 dysregulation has been observed in various cancers, and the signalling it mediates is significantly associated with tumour cell migration, invasion, metastasis and immune regulation.^49^, ^50^ In glioblastoma, high CD99 expression has been found to promote tumour cell migration, invasion and the formation of an immune‐suppressive microenvironment.^51^, ^52^ Overactive activation of the EGF signalling pathway is a crucial feature in glioblastoma, playing a pivotal role in the progression of the disease.^53^ Neoangiogenesis is a significant feature of tumours, and the excessive activation of the VEGF signalling pathway plays a crucial role in this process.^54^ Furthermore, the differing transcription factor activation and metabolic pathway profiles in the high‐ and low‐risk cell groups also underscore the intrinsic distinctions between the two groups. Notably, we observed that high‐risk cell groups exhibited enhanced purine and fatty acid metabolism compared to the low‐risk group. It is worth highlighting that the most heavily weighted molecule in the risk score, SLC43A3, has previously been reported to be associated with the transmembrane transport of purines and fatty acids.^39^, ^40^, ^55^ This suggests that the disparities between the two groups may indeed be attributed to variations in SLC expression patterns. Finally, we performed immunohistochemistry and immunofluorescence staining on glioblastoma samples from the high‐ and low‐risk score groups, confirming the higher expression of CD99, EGFR and VEGFA in the high‐risk group. Interestingly, co‐localization analysis of immunofluorescence for SLC43A3, VEGFA and CD68 has confirmed that high expression of the orphan protein SLC43A3 (the molecule with the highest weight in determining the expression pattern of SLCs) is a significant characteristic of TAMs subpopulation that promotes tumour angiogenesis.

Given the predominant weight of SLC43A3 in determining SLC expression patterns, we further explored the role of SLC43A3 in glioblastoma. Orphan SLC protein SLC43A3 has been relatively understudied and was previously reported to be associated with fatty acid and purine metabolism, serving as a biomarker for chemotherapy effectiveness in lung cancer.^39^, ^55^, ^56^ However, its function in glioblastoma remains unclear. Firstly, we observed a strong positive correlation between SLC43A3 and risk scores, with patients exhibiting high SLC43A3 expression also demonstrating poorer prognosis. Importantly, enrichment analysis consistently revealed the activation of pathways significantly enriched in the high‐risk group, with the high SLC43A3 expression group also showing significant enrichment of the PI3K‐AKT signalling pathway. In glioblastoma, receptor tyrosine kinases such as EGFR and VEGFR activate the PI3K‐AKT signalling pathway via tyrosine residue autophosphorylation, playing a pivotal role in tumour proliferation and progression.^45^ Notably, in our cellular communication analysis, cells with high‐risk scores exhibited enhanced EGF and VEGF signalling pathway network activity. To further investigate the role of SLC43A3 in glioblastoma, we initially assessed SLC43A3 expression at both mRNA and protein levels in glioblastoma cell lines and clinical samples, confirming high SLC43A3 expression in tumour tissues. Subsequently, knockdown and overexpression experiments validated that SLC43A3 promotes glioblastoma cell proliferation and migration. This suggests that SLC43A3 may function as an oncogene in glioblastoma and could potentially serve as a novel therapeutic target for glioblastoma treatment in the future.

However, our study has several limitations. Firstly, we only conducted further experiments on the function of SLC43A3, which exhibited the highest level of weight among the seven SLCs we investigated. The functions of the remaining six SLCs remain to be studied. Additionally, the mechanisms underlying the association of EGFR, CD99 and VEGFA with specific SLC expression patterns were only examined through immunofluorescence or immunohistochemistry in the high‐ and low‐risk groups. Further research is needed to elucidate these mechanisms. Given the unique marking function of SLC43A3 in pro‐angiogenic TAMs, the role of SLC43A3 in TAMs also warrants further exploration. Lastly, whether the impact of SLC43A3 on tumour cell proliferation and migration is related to the PI3K‐AKT signalling pathway requires further experimental confirmation.

## 5 CONCLUSION

In summary, we conducted a comprehensive survey of the genetic landscape of SLCs, established a scoring system based on seven core SLCs and integrated it with other clinical and pathological characteristics to develop a nomogram for predicting the prognosis of glioma patients. Patients in the high‐scoring group exhibited poorer prognoses, a more immunosuppressive microenvironment and varying responses to immunotherapy and chemotherapy agents. Therefore, based on our model, it may be possible to guide patients in different scoring groups towards tailored chemotherapy or immunotherapy regimens. Furthermore, we investigated the relationship between the expression patterns of specific SLCs and their association with malignant cells and TAMs at the single‐cell resolution, particularly in the context of cellular metabolism, transcriptional regulation and cell communication. Finally, we demonstrated the oncogenic role of the orphan protein SLC43A3 in glioblastoma, suggesting its potential as a novel therapeutic target.

## AUTHOR CONTRIBUTIONS

**Wenjie Wu:** Data curation (equal); formal analysis (equal); investigation (equal); methodology (equal); software (equal); validation (equal); visualization (equal); writing – original draft (equal). **Cheng Jiang:** Data curation (equal); formal analysis (equal); investigation (equal); methodology (equal); software (equal); validation (equal); visualization (equal); writing – original draft (equal). **Wende Zhu:** Conceptualization (equal); funding acquisition (equal); methodology (equal); project administration (equal); resources (equal); supervision (equal); writing – review and editing (equal). **Xiaobing Jiang:** Conceptualization (equal); methodology (equal); project administration (equal); resources (equal); supervision (equal); writing – review and editing (equal).

## FUNDING INFORMATION

This work was supported by the Free Innovation Fund of Wuhan Union Hospital (2021xhyn108), Hubei Province Key Laboratory Open Fund for Biologically Targeted Therapy Research (2023swbx023) and National Natural Science Foundation of China (NSFC) (82303127).

## CONFLICT OF INTEREST STATEMENT

The authors declare that the research was conducted in the absence of any commercial or financial relationships that could be construed as a potential conflict of interest.

## Supporting information


Figure S1

Figure S2

Figure S3

Figure S4

Figure S5

Table S1

## Data Availability

The datasets utilized in this study are available online, as described in the Methods section.
