# Supplementary figures and images for: Multi‐omics analysis reveals the association between specific solute carrier proteins gene expression patterns and the immune suppressive microenvironment in glioma
Source: J Cell Mol Med. 2024 Apr 30;28(9):e18339. doi: 10.1111/jcmm.18339 (PMC11060081; doi:10.1111/jcmm.18339)

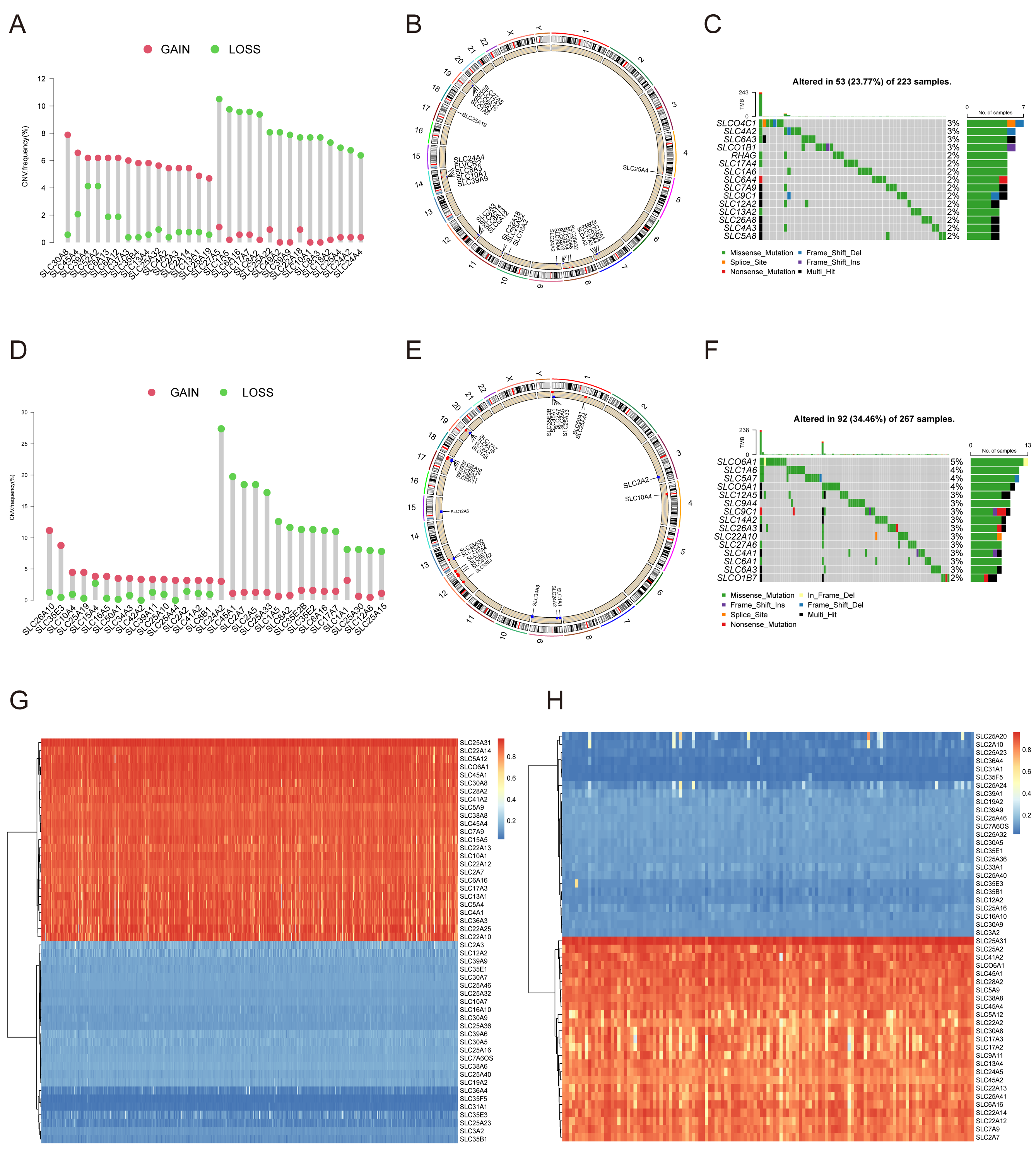

Supplement: Supplementary file 1 — Figure S1 [file JCMM-28-e18339-s005.tif]

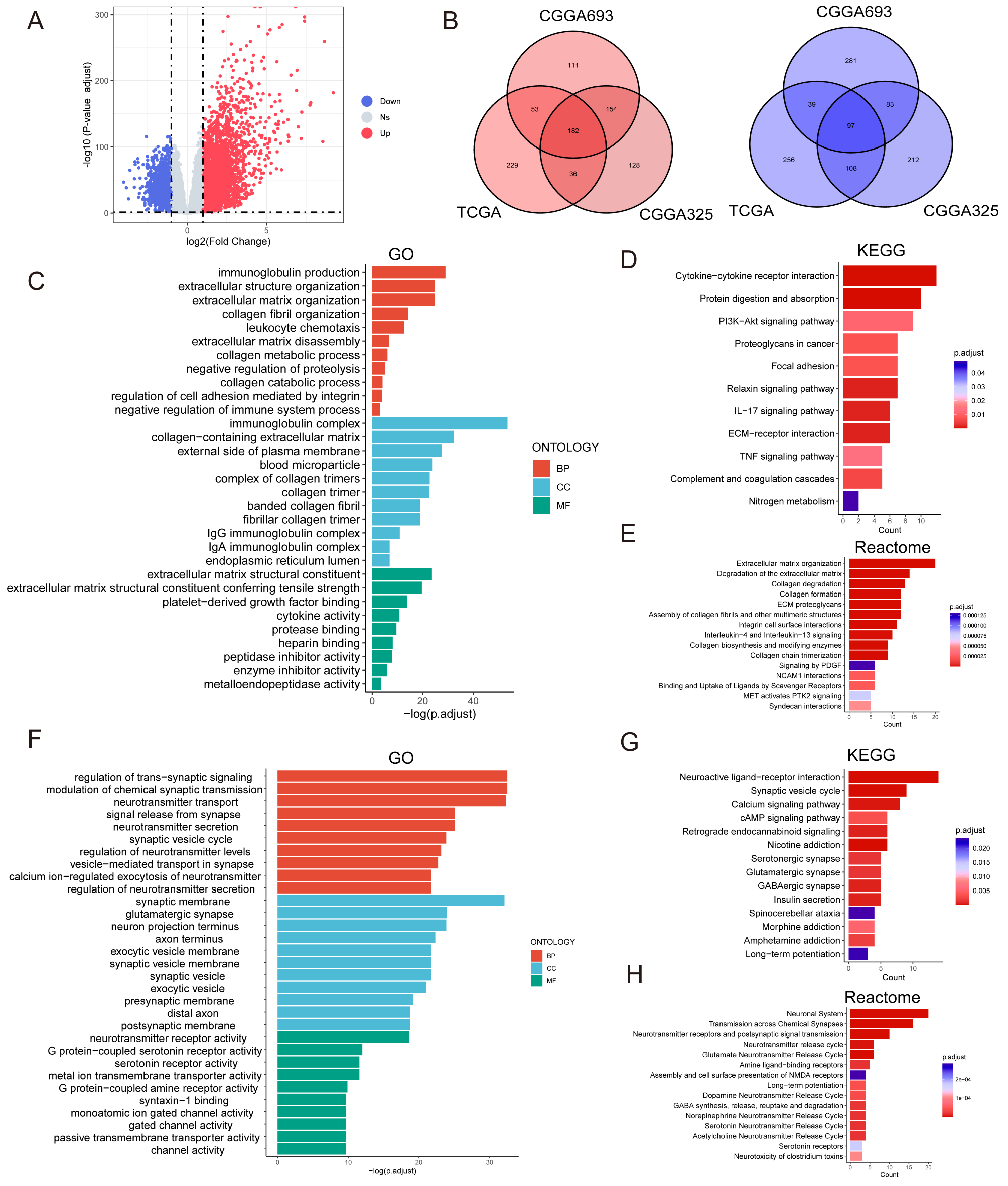

Supplement: Supplementary file 2 — Figure S2 [file JCMM-28-e18339-s002.tif]

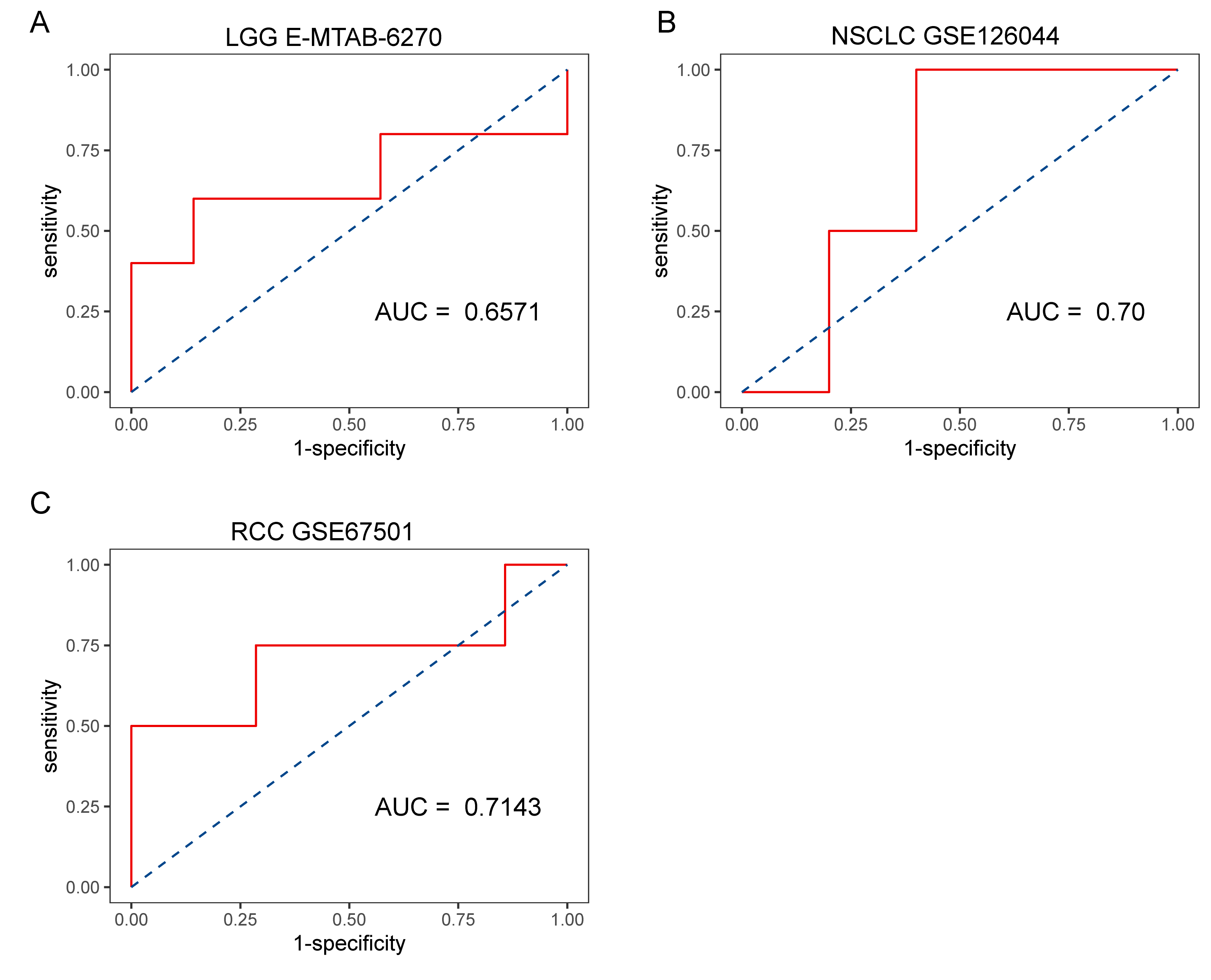

Supplement: Supplementary file 3 — Figure S3 [file JCMM-28-e18339-s004.tif]

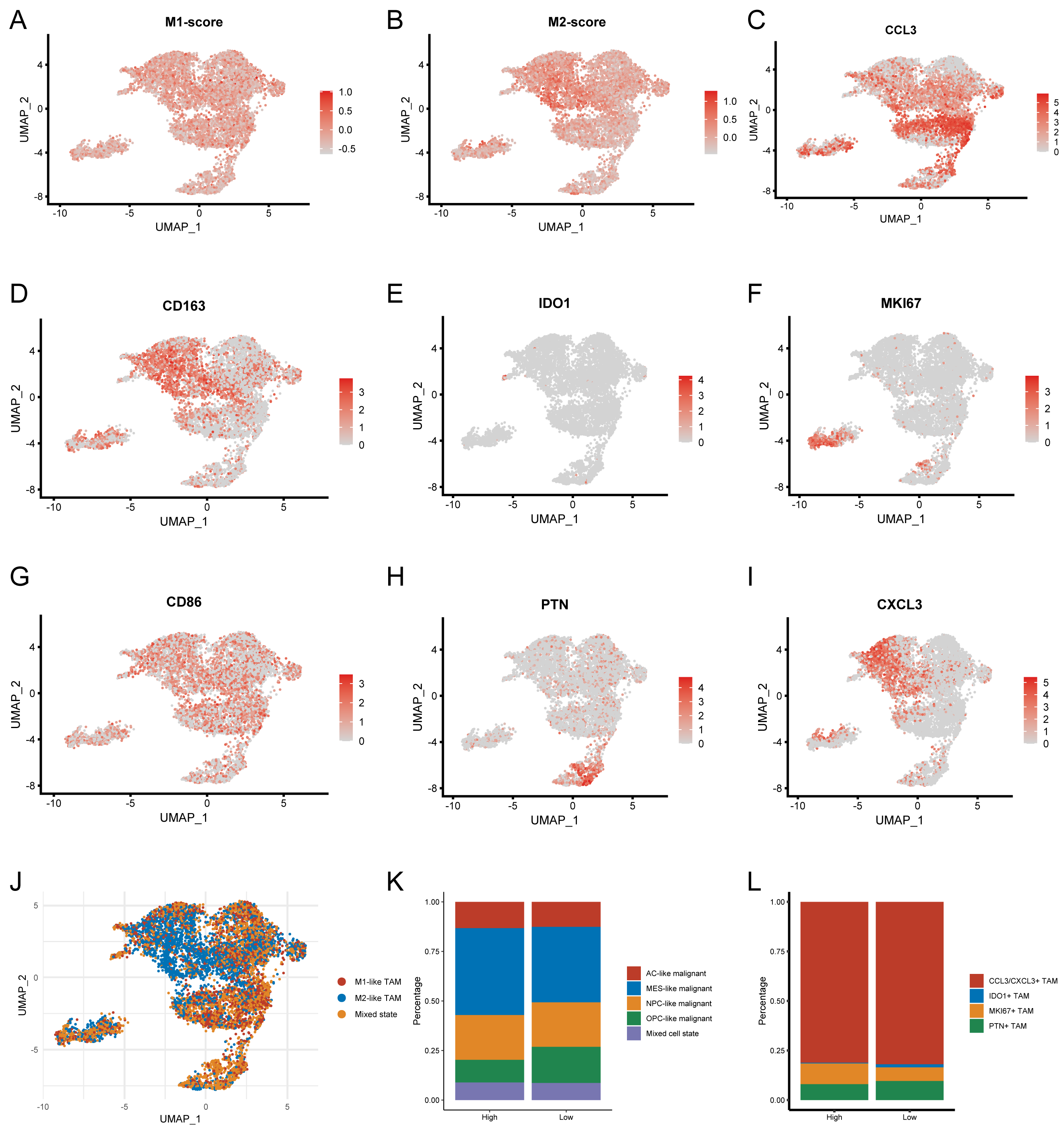

Supplement: Supplementary file 4 — Figure S4 [file JCMM-28-e18339-s006.tif]

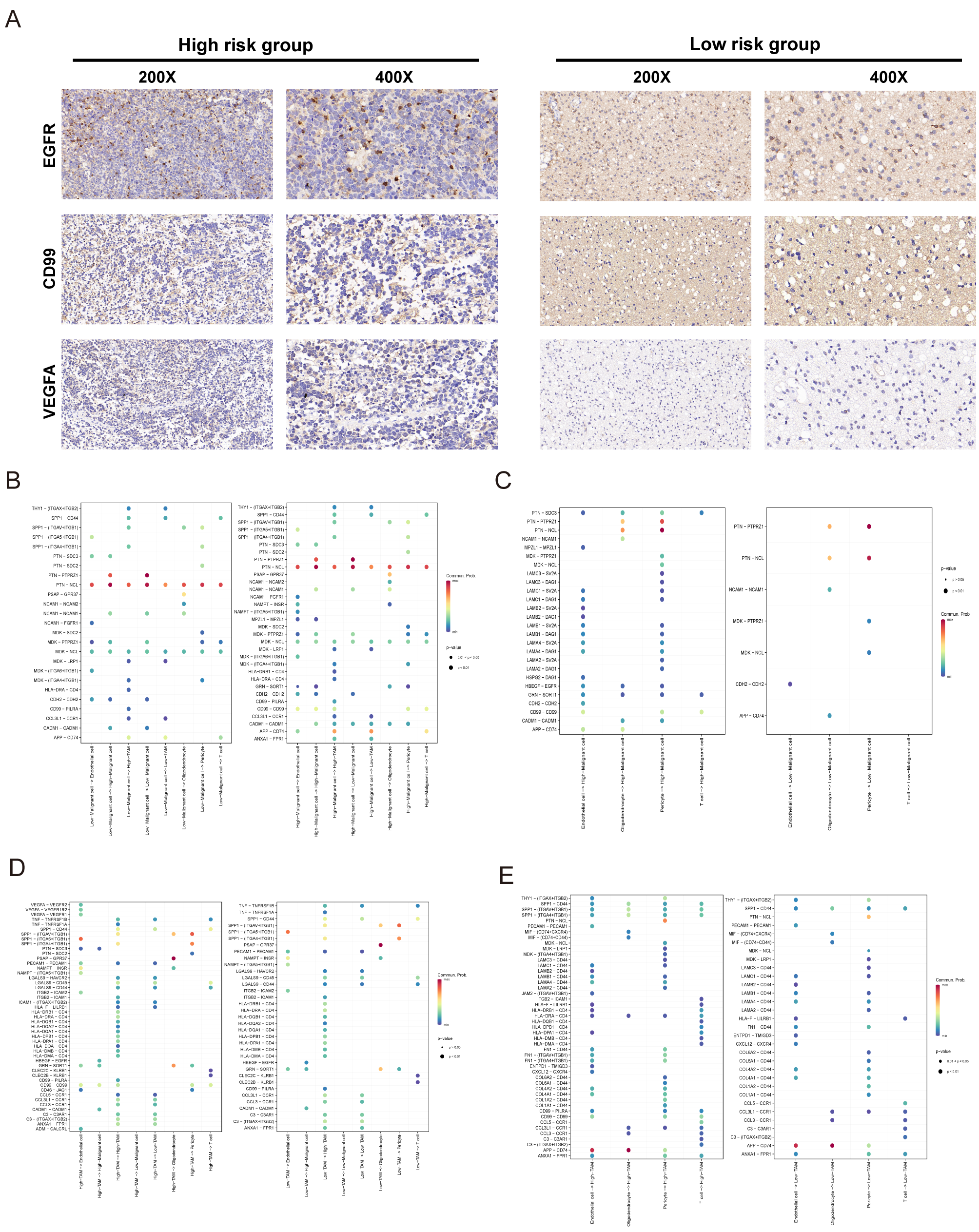

Supplement: Supplementary file 5 — Figure S5 [file JCMM-28-e18339-s003.tif]
